# DrugForm-TAS: Target-Agnostic Selectivity as Proteome-wide Binding Propensity Estimation

**DOI:** 10.34133/csbj.0034

**Published:** 2026-05-18

**Authors:** Anna Tashchilova, Ivan Khokhlov, Alexey Seikin, Nickolay Bugaev-Makarovskiy, Olga Glushkova, Vladimir Yudin, Anton Keskinov, Sergey Yudin, Dmitry Svetlichnyy, Veronika Skvortsova

**Affiliations:** ^1^ Federal State Budgetary Institution “Centre for Strategic Planning and Management of Biomedical Health Risks” of the Federal Medical Biological Agency (Centre for Strategic Planning of FMBA of Russia), Moscow 119121, Russia.; ^2^ The Federal Medical Biological Agency (FMBA of Russia), Moscow 123182, Russia.

## Abstract

Assessing the selectivity of binding a small molecule with proteins remains a fundamental yet unresolved challenge in computational drug discovery. Conventional strategies for estimating ligand selectivity depend on exhaustive cross-prediction of binding affinities across thousands of potential off-target proteins—an approach that is computationally prohibitive for large-scale virtual screening or de novo molecular design. Here, we present DrugForm-TAS (Target-Agnostic Selectivity), the first model capable of directly predicting an unconditional, quantitative measure of small-molecule proteome-wide binding propensity without requiring any prior knowledge of target proteins or affinity thresholds. Built upon our previously developed DrugForm-DTA model, which enables affinity prediction for arbitrary protein targets, and trained on a thoroughly curated dataset derived from BindingDB, DrugForm-TAS employs a lightweight transformer-like neural network that operates solely on a ligand’s SMILES representation. The model acts as a fast pre-screening filter that can greatly reduce the candidate set, as evidenced by the correlation between its predictions and experimental observations. By eliminating the need for target-specific computations, DrugForm-TAS enables target-agnostic nonspecificity profiling. When combined with drug–target affinity calculation, it provides a novel tool for fast pre-screening selectivity estimation in early-stage drug design.

## Introduction

The development of a new drug is an expensive and labor-intensive process. On average, it takes 10 to 20 years and costs over US$1 billion to bring a drug to market [1,2]. Recent advances in computational methods and technologies—including the application of machine learning (ML), particularly deep learning, to chemical and biological research—enable simultaneous expansion of the search space and reduction in the number of candidate lead molecules. Methods for predicting the binding affinity between a small molecule and a protein (drug–target affinity [DTA]) have become widely adopted and are especially valuable for high-throughput screening of drug-like compound libraries and for the de novo design of novel ligands.

Currently, developers of DTA models have made substantial progress, and modern models are capable of effectively predicting binding affinities between ligands and proteins [3–5]. However, despite substantial efforts to improve every aspect of the drug development process over the past few decades, the clinical success rate of drug development remains at approximately 10% to 15% [1]. One of the primary reasons for this low success rate is the occurrence of side effects. Several studies have developed ML models for predicting adverse drug reactions [6–8]. One of the most widely used datasets for training such models is SIDER, which contains 1,427 molecules and 27 classes of adverse effects defined by the Medical Dictionary for Regulatory Activities [9]. Existing side-effect datasets are limited in size and scope, making it difficult to train robust models. Moreover, adverse effects can arise from a variety of factors, including drug–drug interactions, individual intolerance—such as toxicity and/or excessive immune responses—in specific patients, and other biological or environmental influences [10]. Thus, developing an algorithm to predict the side effects caused by an approved drug using limited data remains an exceptionally ambitious challenge.

However, some adverse effects can be attributed to the binding of the active substance of the drug to off-target proteins, and DTA models have proven highly effective in addressing this issue. A model that predicts such off-target interactions would facilitate the identification of target-selective ligands during the computational design stage.

Assessing the potential number of off-target binding sites during the in silico development of an active compound would enable preliminary filtering of candidate molecules for experimental in vitro and in vivo testing. This assessment can be performed using DTA models. The approach involves proteome-wide prediction of the binding affinity of each ligand against all target proteins and counting the number of off-target interactions based on a predefined threshold. However, contemporary DTA models are typically highly complex and computationally demanding. Consequently, the requirement to evaluate each molecule against thousands of proteins renders large-scale screening of millions of drug-like small molecules for off-target effects impractical or infeasible.

A step toward addressing the challenge of large-scale small-molecule screening was proposed in an approach [11] for assessing target-specific selectivity in drug development and repurposing. The method was developed using the Davis dataset [12] and enabled discrimination between highly selective and multitarget kinase inhibitors through the *k*-nearest neighbors algorithm. The study introduced a selectivity metric defined as a weighted sum of global and local relative potencies. Global relative potency is more robust to missing data than local relative potency. The method can identify compounds that are selective despite exhibiting relatively low binding affinity. This approach reduces the number of targets for which affinity predictions are required.

An approach to assessing off-target effects during de novo antiviral ligand design using DTA prediction is implemented in the CogMol framework [13]. CogMol is a generative platform for designing novel small-molecule antivirals with high target affinity and selectivity, combining adaptive pre-training of a Simplified Molecular Input Line Entry System (SMILES)-based [14] molecular variational autoencoder [15] with an efficient supervised, multiobjective sampling strategy. In CogMol, selectivity is quantified as the difference between a molecule’s predicted affinity for the target protein and its mean affinity across *k*-randomly selected off-target proteins—a strategy conceptually similar to that described by Wang et al. [11].

Although the considered methods improve upon simultaneous affinity prediction across all proteins, they still require computationally expensive pre-prediction of ligand affinities for a subset of off-target proteins. Furthermore, reducing the number of targets compromises the accuracy of selectivity estimates. This requirement on a protein subset stems from the prohibitive computational cost of evaluating ligand affinities against every protein. Moreover, these approaches assume a known target protein for the ligand under study.

Developing a method to assess selectivity without requiring proteome-wide prediction of ligand affinity against every protein remains a critical challenge. Moreover, integrating the selectivity model into the generative pipeline would reduce the production of ligands with off-target binding. Moreover, it could accelerate large-scale virtual screening by estimating the selectivity of a single small molecule in a time comparable to that of a single affinity prediction.

Here, we introduce a fundamentally new approach for the computationally efficient estimation of small-molecule off-target bindings. We developed a deep learning model—DrugForm-TAS (Target-Agnostic Selectivity)—that takes a ligand as a SMILES string and outputs a set of continuous values between 0 and 1. The model provides both threshold-specific selectivity scores (at affinity thresholds 7.0, 7.5, 8.0, and 8.5) and a unified, threshold-agnostic estimate of off-target binding propensity. Critically, DrugForm-TAS does not require a predefined target protein, as it evaluates proteome-wide binding propensity independently of any reference target—hence the name Target-Agnostic Selectivity. The primary application of the model is fast large-scale pre-screening of small-molecule libraries to prioritize its affinity to different target proteins. Additionally, DrugForm-TAS can be integrated into de novo ligand design pipelines to suppress off-target binding during molecular generation.

## Materials and Methods

### Neural network architecture

**Fig. 1.**
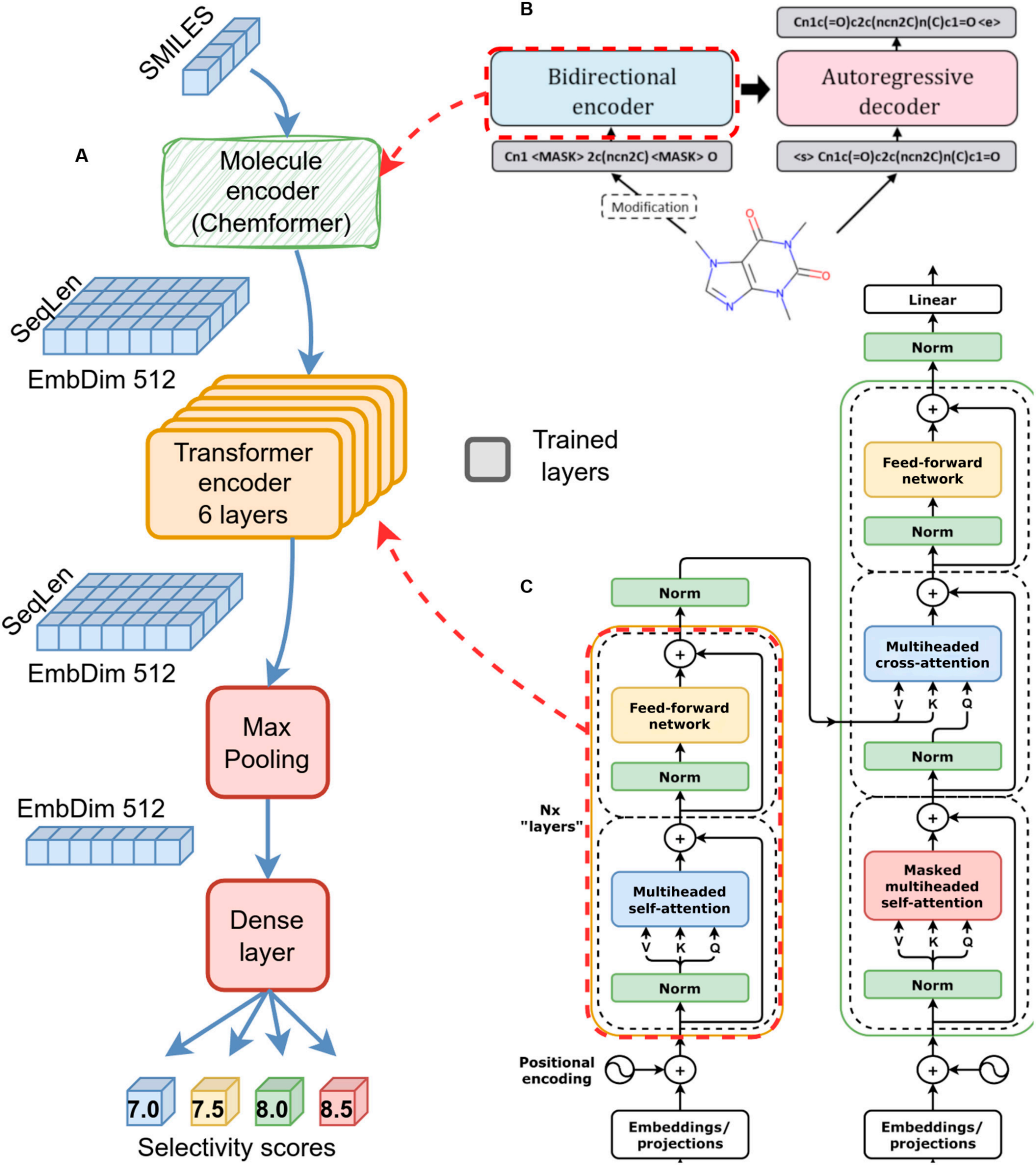
DrugForm-TAS neural network architecture. (A) DrugForm-TAS neural network architecture. The encoder block takes the molecular embedding from the Chemformer model as input. A MaxPooling layer aggregates the transformer’s internal representations into a fixed-size vector, which a linear layer then maps to the output selectivity scores. (B) Using the Chemformer model (combined variant) as a nontrainable molecule embedding generator. Adopted from [17]. (C) The original transformer-encoder architecture. Reproduced from [16].

Transformer-based neural networks are widely used in various ML tasks, allowing achievement of outstanding results in different areas of computer science: natural language processing, computer vision, etc. The original transformer was presented by Vaswani et al. [16] as a tool for solving the machine translation task (Seq2Seq). Transformer-based neural networks effectively process string sequences of different lengths, including SMILES representations of ligands. Thus, they become a standard tool in chemoinformatics. This study does not introduce a novel neural network architecture. Instead, we adopt a functional and constructive approach including a molecule encoder pre-trained on a large number of chemical structures, followed by a trainable transformer encoder (Fig. 1).

The DrugForm-TAS neural network architecture (Fig. 1A) uses the pre-trained molecular encoder Chemformer [17] (combined variant, Fig. 1B) that has already demonstrated strong performance as an embedding generator in DrugForm-DTA. Chemformer is a transformer-based model based on the bidirectional autoregressive transformer [18], a combination of BERT (bidirectional encoder representations from transformers) [19] and a transformer decoder.

Chemformer-derived numerical representations of small molecules are passed to a vanilla 6-layer transformer encoder (Fig. 1C). To obtain a fixed-size output from the transformer’s internal representation, we apply MaxPooling—a dimensionality-reduction operation that computes the maximum activation across sequence positions—followed by a single-layer dense layer. The embedding dimension is 512 in all layers, following the Chemformer dimension.

### DrugForm-DTA

Protein–ligand affinities were predicted using the DrugForm-DTA model [20]. DrugForm-DTA takes as input text representations of the protein (as an amino acid sequence) and the ligand (as a SMILES string). The model employs a transformer-based neural network architecture, with protein encoding based on the ESM (Evolutionary Scale Modeling) model [21] and ligand encoding based on the Chemformer model [17]. We evaluated the neural network architecture on the Davis [12] and KIBA [22] benchmark datasets, where it demonstrated state-of-the-art performance. For practical application, we trained the DrugForm-DTA model on a curated version of the BindingDB database [23]. This training dataset was constructed through multistep data filtering and aggregation and includes a weight reflecting the importance of each ligand–protein pair. The final dataset comprises 1,739,873 ligand–protein pairs (5,251 proteins and 1,020,614 ligands) with associated amino acid sequences, SMILES, pKi, pIC50, and weights. It is freely available at https://doi.org/10.5281/zenodo.14949569.

In this study, we used the curated BindingDB dataset to construct the training set for the target-agnostic selectivity (TAS) model. We also used DrugForm-DTA to calculate affinities between each ligand and all proteins for the correlation analysis.

### Dataset for TAS model

A training selectivity model requires a dataset containing affinity values for each ligand to as many targets as possible. In practice, experimenters study the affinity of a ligand for a small group of target proteins of their interest. This leads to a dramatically sparse ligand–protein interaction table, but we need the model to learn interactions with a different variety of proteins for each target. Thus, we decided to calculate binding affinity with the DrugForm-DTA model [20].

First, we took the BindingDB dataset, prepared in the DrugForm-DTA work, and took all unique ligands (1,020,614) from it. From the UniProt database [24], we extracted all human proteins (20,417) and retained 2,103 unique proteins that overlapped with BindingDB.

Second, we calculated the affinity for each unique ligand to all protein targets. The DrugForm-DTA model predicts pKi and pIC50 separately. In practice, they are close numerically, although they have different nature. In this work, we require a single affinity value, so we decided to average affinity values as (pKi + pIC50)/2. Despite the differences in the nature of these parameters, in this problem there is no need to predict or interpret their average value; only an aggregate value is required, which will be compared numerically with similar values. For this reason, we consider such averaging justified in this context.

Third, for each ligand, we counted the proteins with affinity values exceeding each of the 5 predefined thresholds—7.0, 7.5, 8.0, 8.5, and 9.0—and got 5 numbers for each ligand. We calculated average counts for each threshold among all ligands and used them to binarize count values to selectivity scores, which are inversely proportional to the number of proteins to which a given ligand binds. Thus, we got a table, where for each ligand and for each threshold a binary selectivity score is assigned: 0 indicating low selectivity and 1 indicating high selectivity. The binarization was performed using mean binding counts for each of 5 columns: 195, 49, 8, 1, and 0.

Furthermore, for each ligand, we assigned a weight reflecting its training importance: a higher weight was given to rarer selectivity profiles. First, we normalized each column by its mean, yielding per-column weights. The weights from the 4 columns were then averaged to produce a single weight per molecule. Normalizing this vector by its mean yielded the final sample weights. The distribution has a mean of 1.0, a standard deviation of 1.2, and a maximum-to-minimum ratio of 10.9, indicating a reasonably balanced weight distribution. This mitigates training instability associated with class imbalance.

Incorporating these weights helped us decrease class imbalance. Our analysis of the resulting dataset revealed that affinity values exceeding the 9.0 threshold were too sparse to train a robust model, as 98% of entries were zeros. Consequently, we excluded this threshold from the final dataset.

The final training dataset contains the following features: SMILES; binarized selectivity labels at thresholds 7.0, 7.5, 8.0, and 8.5; and sample weights. We split the dataset into training and test subsets: the test set containing 5% (50,829 molecules) and the training set containing 969,771 molecules.

### Dataset for correlation test

The TAS model was trained on artificial data, first-order derivative from experimental values. In order to understand how much affinity information was lost, we performed a massive test, comparing 3 different selectivity estimation approaches: this model, DrugForm-DTA, and directly obtaining from experimental values. Obtaining selectivity scores from the TAS model requires just ligand itself. To calculate selectivity scores from a DTA model, we need to get the ligand affinity for each protein. These values already exist in the training dataset. Estimating selectivity from experimental values is a more challenging task. We suggest calculating it as the proportion of affinity values exceeding a threshold among all records with known experimental values.

The same dataset is required for each of these 3 methods. We construct the dataset for the selectivity model correlation test from the BindingDB dataset, prepared in the DrugForm-DTA work [20], selecting ligands that have at least 10 experimental measurements of affinity constants for different proteins. The resulting dataset contains 4,298 ligands satisfying this criterion and includes measured affinity values for 1,769 unique human proteins.

The resulting correlation test dataset consists of 4,298 SMILES strings, for which we computed selectivity scores for 4 thresholds—7.0, 7.5, 8.0, and 8.5—using each of the 3 methods: DrugForm-TAS, DrugForm-DTA, and the experimental approach.

### Model quality metrics

A confusion matrix is used in ML to evaluate the model performance on a binary classification task. In such tasks, samples are labeled as either positive (1) or negative (0). Consequently, each prediction falls into 1 of 4 categories: true positive (TP), false positive (FP), true negative (TN), or false negative (FN). The confusion matrix—a 2 × 2 contingency table reporting the counts of TP, FP, TN, and FN—enables the computation of multiple evaluation metrics, including accuracy, precision, and recall.

Unlike standard accuracy, which can be deceptively high in imbalanced datasets, balanced accuracy provides a more informative assessment by incorporating both precision and recall. This metric is particularly suited for class-imbalanced scenarios and is defined as

$$\text{Balanced accuracy}=\frac{\text{Precision}+\text{Recall}}{2}$$ (1)

where

$$\text{Precision}=\frac{\mathrm{TP}}{\mathrm{TP}+\mathrm{FP}},\;\text{Recall}=\frac{\mathrm{TP}}{\mathrm{TP}+\mathrm{FN}}$$ (2)

The F1-score is the harmonic mean of precision and recall [25]. This metric is commonly used in class-imbalanced settings and is defined as

$$F1=\frac{2\times \text{Precision}\times \text{Recall}}{\text{Precision}+\text{Recall}}$$ (3)

The performance of the DrugForm-TAS model was illustrated using the F1 curve, which shows F1-scores across all possible classification thresholds. The F1 curve shows the trade-off between FPs and FNs at varying thresholds. The precision–recall curve is used to evaluate classifier performance under class imbalance and illustrates the trade-off between precision and recall at different classification thresholds.

The performance of the classification model across all possible classification thresholds was also illustrated using a receiver operating characteristic (ROC) curve. The false positive rate (FPR) is plotted on the *X*-axis and the true positive rate (TPR) on the *Y*-axis: TPR = TP/(TP + FN) and FPR = FP/(FP + TN). The ROC curve graphically shows the trade-off between precision and recall at varying classification thresholds. An ideal classifier would approach the upper left corner of the plot, where TPR = 1 and FPR = 0.

The area under the curve (AUC) summarizes model performance as a single scalar value ranging from 0 to 1, with higher values indicating superior discriminative ability.

### Training procedure

The DrugForm-TAS model is trained to predict all threshold selectivity values simultaneously. Since the 7.0, 7.5, 8.0, and 8.5 threshold selectivity values correlate, training one of the parameters improves the prediction of the other parameters. The prepared dataset was divided into training (95%, 969,771) and test (5%, 50,829) subsets, and the test subset did not participate in the training procedure in any role.

Cross-validation (CV) training is used to prevent any risk of overfitting and to obtain a robust model. The training subset was divided into training and validation subsets in 4 parts without crossing the validation sets (classical *K*-fold CV [26]). Each submodel learned on three-fourths of the full training set and validated at one-fourth of the full training set. The validation subset is used as overfitting control and model selection criterion. This approach improves the predictive power of the model at the cost of additional computations.

The neural networks used in the model are computationally heavy, but the embeddings produced by the nontrainable ligand encoder do not change over time and were cached. Thus, the additional cost of introducing 4 submodels is less than 4 times. At inference, the step outputs of all submodels are averaged.

Thus, 4 submodels were trained on their own CV splits. It took 2 d to train each submodel at an NVidia A100 graphics processing unit (GPU). Architecture and training parameters were identical for each submodel, including 6 encoder layers with depth 512, 1 encoder layer, ReLU activation, RAdam optimization procedure with learning rate 0.003, batch size 128, and early stopping with up to 100 epochs. Full details on the architecture and training procedure is available in the training code (see Data Availability). Each of the 4 submodels contains 37 million trainable parameters.

All program code is written in Python using PyTorch framework version 1.13 [27]. The implementation of transformer layers is taken from the fairseq library [28]. We also used RDKit v2023.9.5 and MolVS v0.1.1 to process and standardize SMILES.

## Results

### Developing a training dataset for the DrugForm-TAS model

The training of a selectivity model requires affinity predictions for each ligand against all proteins of interest. Since no experimentally derived affinity dataset of sufficient scale exists for this purpose, we constructed a novel dataset based on the BindingDB dataset. We used the preprocessed BindingDB dataset from DrugForm-DTA [20], which was curated for neural network training through multi-stage filtering and aggregation.

**Fig. 2.**
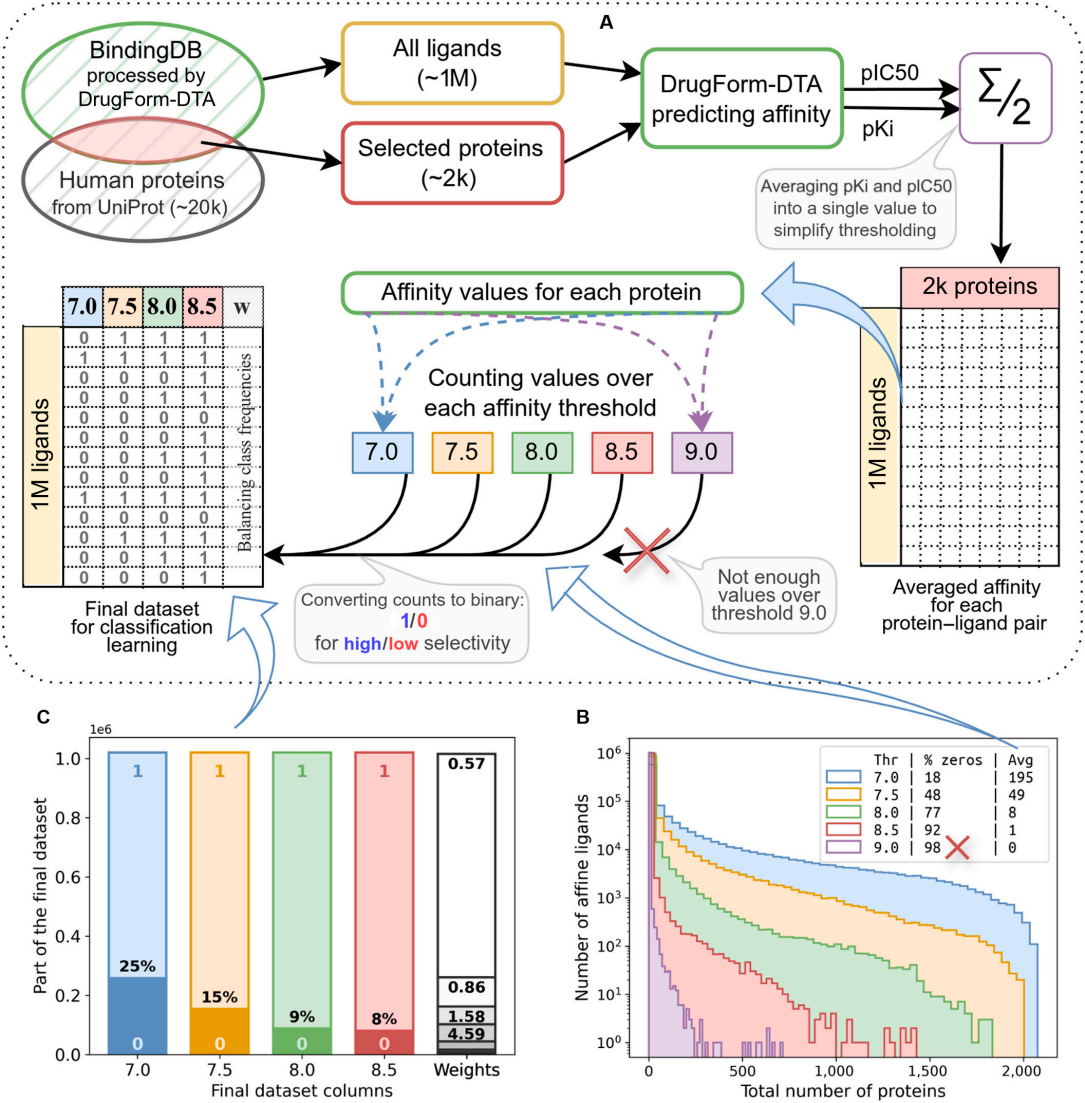
The DrugForm-TAS model training dataset construction. (A) Schematic diagram of the training dataset preparation. We used the DrugForm-DTA affinity prediction model to identify target proteins for each ligand in the BindingDB database at multiple affinity thresholds. The number of predicted targets per ligand was binarized to generate selectivity labels. The final dataset contains approximately one million ligands, each annotated with binary selectivity scores at thresholds of 7.0, 7.5, 8.0, and 8.5, along with a sample weight for training. (B) Histogram showing the distribution of human proteins by the number of ligands predicted to bind them. For each threshold, we report the mean number of binding ligands per protein and the part of proteins with no predicted binders. The “Avg” column contains mean values for each threshold, used as a binarization threshold while constructing the training dataset. (C) Distribution of selectivity labels in the final training dataset.

The procedure for constructing the training set (Fig. 2A) is described in detail in the materials and methods section. For each ligand in the preprocessed BindingDB dataset, we derived qualitative selectivity estimates that are inversely proportional to the number of proteins to which the ligand binds. We excluded the 9.0 threshold because 98% of ligands showed no predicted binding above this level, precluding the assembly of a statistically meaningful training set (Fig. 2B). For the remaining thresholds, we performed binarization by average values (Fig. 2B) and generated binary selectivity labels (Fig. 2C): 25% of ligands were classified as nonselective at threshold 7.0; 15%, at 7.5; 9%, at 8.0; and 8%, at 8.5.

The resulting dataset exhibited class imbalance, which we addressed by assigning a sample weight to each ligand. Weights were obtained according to procedure, described in Materials and Methods. We used these weights during training, with higher weights assigned to ligands exhibiting rarer selectivity profiles. The final training dataset contains 1,020,614 ligands, each annotated with binary selectivity labels at thresholds 7.0, 7.5, 8.0, and 8.5, as well as a corresponding training weight.

The resulting dataset of off-target binding predictions is freely available (see Data Availability) and can be used to train next-generation selectivity models. It is the largest of its kind to date, comprising affinity predictions for over 2.1 billion protein–ligand pairs. These computations were carried out on 8 NVIDIA A100 GPUs over a 3-month period.

### Training and performance of the DrugForm-TAS model

**Fig. 3.**
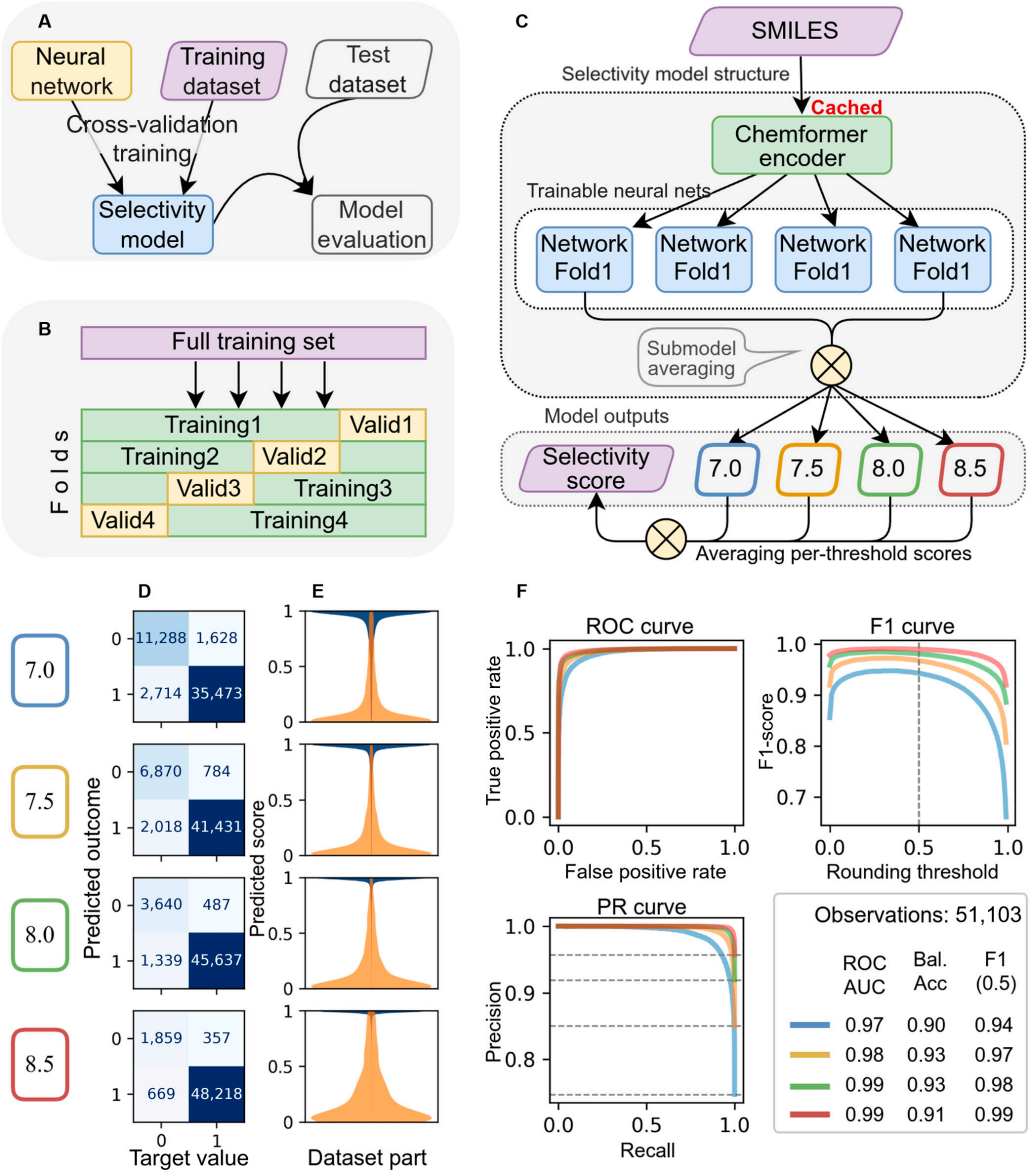
Training and evaluation of the DrugForm-TAS model. (A) Splitting the original dataset into training and test subsets. (B) Splitting the training dataset into a group of training and validation subsets by *K*-fold cross-validation: individual parts of the dataset alternately become training or validation. (C) Structure of the Target-Agnostic Selectivity (TAS) model. The model consists of 4 submodels, 1 for each cross-validation fold. The results of the submodels are averaged to obtain a consolidated selectivity value at each threshold (7.0, 7.5, 8.0, and 8.5). These are further averaged to produce a single threshold-agnostic selectivity estimate. Thus, the model takes a Simplified Molecular Input Line Entry System (SMILES) string as input and outputs 5 scalar values: 4 threshold-specific scores and 1 threshold-agnostic score. (D) Confusion matrices for the DrugForm-TAS model on the test subset. (E) Distribution of raw model outputs prior to binarization. (F) Classification metrics (ROC-AUC, balanced accuracy, F1-score, precision, and recall) at each threshold. ROC, receiver operating characteristic; AUC, area under the curve.

We split the developed selectivity dataset into training and test subsets. We trained the DrugForm-TAS neural network model on the training subset, while the test subset was not directly or indirectly involved in training (Fig. 3A). Applying the classical *K*-fold CV procedure yielded 4 independently trained submodels, which we combined into an ensemble (Fig. 3B). The training procedure is explained in detail in the materials and methods section. Each submodel outputs selectivity scores for each threshold—continuous scores between 0 and 1 (Fig. 3C). In the inference mode, predictions of each submodel are averaged, producing a single value for each threshold—7.0, 7.5, 8.0, and 8.5.

We evaluated the performance of the trained model on a test subset, which was not involved in training. Confusion matrices (Fig. 3D) summarize the true and false predictions of DrugForm-TAS on the test set for each threshold, distinguishing FNs from FPs. We also visualized the distribution of raw model outputs prior to binarization (Fig. 3E). These distributions show that predictions are typically concentrated near 0 or 1, indicating high-confidence classifications. In contrast, predictions closer to 0.5 reflect greater uncertainty. It could be used for understanding, where we can or cannot trust the model prediction. For the 8.5 threshold, the model shows higher prediction uncertainty than for other thresholds—a consequence of the more severe class imbalance in the corresponding training subset. Performance metrics (Fig. 3F) confirm that the model successfully adopted the training task and the behavior of the parent DTA model as the distillation process: ROC-AUC ranged from 0.97 to 0.99; F1-scores, from 0.94 to 0.99; and balanced accuracy, from 0.90 to 0.93. Since the training dataset had a strong class imbalance, the ROC-AUC values optimistically describe the model performance. We consider the balanced accuracy metric values as the most reliable estimation of the model performance.

Regarding the binarization of model predictions, in practice, the model outputs a continuous value in the range [0, 1], where deviation from the extremes (0 or 1) reflects the model prediction uncertainty. Nevertheless, we computed calibration curves for binarization at each affinity threshold (Fig. S6). For users requiring binary outputs, the optimal decision thresholds (maximizing F1-score) are 0.36, 0.35, 0.27, and 0.33 for thresholds 7.0, 7.5, 8.0, and 8.5, respectively.

Additionally, we computed a threshold-agnostic selectivity score by averaging the 4 threshold-specific outputs, yielding a single global estimate of ligand selectivity that is independent of any target or affinity threshold.

### Calculating target-agnostic selectivity

To assess whether a unified selectivity metric could be derived, we averaged the model’s predictions across all 4 affinity thresholds (7.0, 7.5, 8.0, and 8.5). We then computed Spearman correlations between this averaged score and the threshold-specific predictions, obtaining values of 0.992, 0.993, 0.964, and 0.578, respectively. The averaged score shows very strong agreement with predictions at thresholds 7.0, 7.5, and 8.0 and moderate correlation at the 8.5 threshold.

Given the strong correlations between the averaged selectivity score and all threshold-specific predictions, we formalized the concept of TAS. This approach demonstrates that a single scalar value can effectively characterize a ligand’s off-target binding propensity—without requiring specification of either a target protein or an affinity threshold. To validate this simplification, we computed the Spearman correlation between the model’s threshold-agnostic score and the corresponding selectivity estimate derived from DrugForm-DTA proteome-wide predictions, yielding a correlation of 0.718. This result indicates that the target-agnostic score is meaningfully consistent with cross-target DTA–based selectivity assessments.

It should be emphasized that TAS is not selectivity in the strict pharmacological sense, but rather an integrated estimate of a ligand’s propensity to engage multiple proteins across the affinity range of practical relevance (pKi/pIC50 7.0 to 8.5). Accordingly, the TAS metric is defined as the mean of binary selectivity scores computed at 4 affinity thresholds: 7.0, 7.5, 8.0, and 8.5. We illustrate the utility of DrugForm-TAS through several examples of threshold-free selectivity estimation for representative small molecules.

For example, consider the ligand O=C(O)c1ccc(Nc2ncc3c(n2)-c2ccc(Cl)cc2C (c2c(F)cccc2F)=NC3)cc1 from the test set, which has 65 experimentally measured affinities across diverse proteins. DrugForm-TAS predicts a high selectivity score (TAS = 0.9). Consistent with this prediction, the ligand exhibits strong affinity only for Aurora kinase A (UniProt ID: P97477; pKi = 8.15, pIC50 = 8.40), while its remaining affinities average pKi/pIC50 ≈ 4.6—well below the threshold of pharmacological relevance (typically ≥7.0).

In contrast, the ligand CN[C@@H]1C[C@H]2O[C@@](C)([C@@H]1OC)n1c3 ccccc3c3c4c(c5c6ccccc6n2c5c31)C(=O)NC4, for which 68 experimental affinities are available, receives a TAS score of 0.0, indicating high promiscuity. Indeed, it shows high-affinity interactions with 27 proteins (pKi = 7.1 to 9.9; pIC50 = 5.8 to 10.2), confirming its nonselective binding profile.

Computing the TAS score does not require retraining the model. DrugForm-TAS independently predicts selectivity at 4 affinity thresholds (7.0, 7.5, 8.0, and 8.5), each of which can be used separately. Alternatively, these 4 outputs can be averaged to yield the unified TAS score. The published model performs this aggregation automatically and returns all 5 values (4 threshold-specific scores and the TAS score; see Data Availability). This means that users can choose their own way to average thresholded values into a custom-weighted threshold-agnostic value.

### DrugForm-TAS correlation test

Since the TAS model can be viewed as a distillation of the DTA model, standard evaluation metrics such as ROC-AUC or balanced accuracy should not be interpreted as direct measures of its ultimate utility. Instead, they reflect how well the trained TAS model reproduces the selectivity behavior encoded in the DTA predictions. While this is undoubtedly an important indicator of training success, it only indirectly captures the model’s ability to fulfill its primary objective: providing proteome-wide binding propensity score.

In a perfect world, TAS predictions would be benchmarked against experimental selectivity estimates. Unfortunately, constructing such a dataset is practically infeasible, as it would require measuring the affinity of each ligand against the entire human proteome. Nevertheless, it remains valuable to evaluate the model on available experimental data, even if limited in scope. We therefore curated a subset of BindingDB comprising ligands with a statistically substantial number of experimentally measured affinities across diverse proteins and developed a method to estimate experimental selectivity within this subset. Because direct comparison between proteome-wide and partial-proteome selectivity estimates is not meaningful, we instead assessed the correlation between these metrics.

**Fig. 4.**
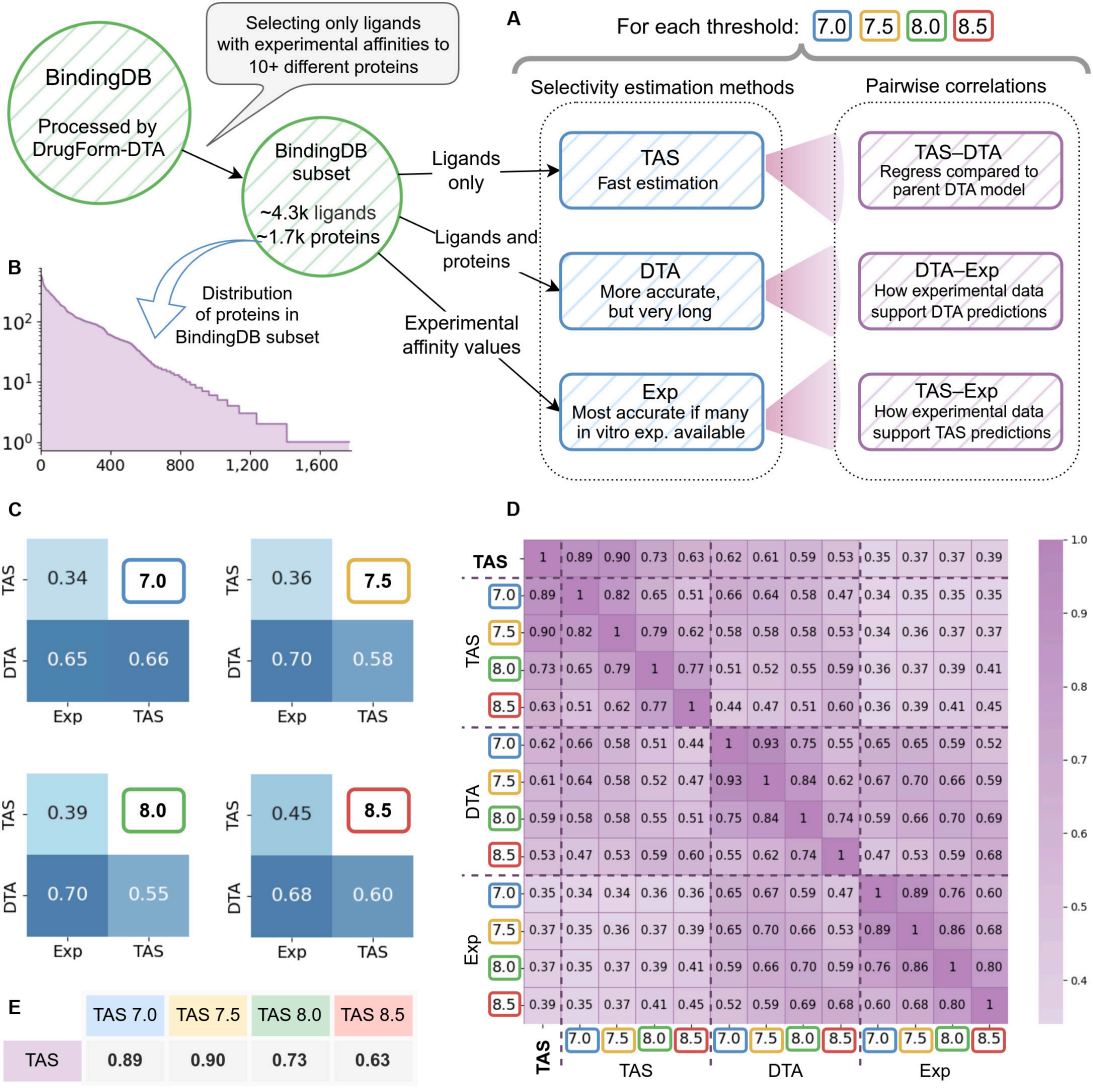
Correlation test of the DrugForm-TAS model. (A) Schematic diagram of the dataset preparation for the correlation test. Ligand selectivity was assessed using 3 approaches: the DrugForm-TAS model, cross-prediction of affinities via DrugForm-DTA, and direct estimation from experimental data. (B) Frequency distribution of the ~4,300 unique human proteins in the correlation dataset. (C) Pairwise heatmaps across the 3 selectivity estimation methods—DrugForm-TAS, DrugForm-DTA, and experimental—for each of the 4 affinity thresholds (7.0, 7.5, 8.0, and 8.5). (D) Overall correlation heatmaps showing Spearman coefficients between all methods (DrugForm-TAS, DrugForm-DTA, and experimental) at thresholds 7.0, 7.5, 8.0, and 8.5, as well as the threshold-agnostic DrugForm-TAS output. (E) Spearman correlation between the threshold-agnostic DrugForm-TAS selectivity score and its threshold-specific scores at 7.0, 7.5, 8.0, and 8.5.

We validated the effectiveness of the DrugForm-TAS selectivity model through a correlation test using a dataset derived from BindingDB (Fig. 4A); see Materials and Methods. We selected ligands with at least 10 experimentally measured affinities across distinct human proteins. The resulting dataset comprises 4,298 ligands satisfying this criterion and includes affinity measurements for 1,769 unique human proteins (Fig. 4B). The number of proteins in the correlation test is smaller than that in the DrugForm-TAS training set (2,103) because we excluded proteins with insufficient ligand coverage for reliable statistical estimation.

We compute selectivity estimates for each ligand in the correlation dataset using 3 distinct approaches (Fig. 4A). First, we used DrugForm-TAS to generate selectivity scores at 4 affinity thresholds (7.0, 7.5, 8.0, and 8.5) and a threshold-agnostic score. Second, we predicted affinities for each ligand against all 2,103 unique proteins in the curated BindingDB dataset using DrugForm-DTA. The DTA-based selectivity estimate was defined as the fraction of predicted affinities exceeding a given threshold relative to the total number of proteins. Third, we derived selectivity estimates from experimental affinity data in BindingDB, calculating the fraction of measured affinities above each threshold relative to the total number of experimentally assayed proteins for that ligand.

We computed pairwise Spearman correlations among the 3 selectivity estimation approaches—DrugForm-TAS, DrugForm-DTA, and experimental—for each of the 4 affinity thresholds (Fig. 4C). The strongest correlations were observed at thresholds 8.0 and 8.5. A comprehensive comparison across all methods and thresholds, including the threshold-agnostic DrugForm-TAS output (Fig. 4D), revealed that DrugForm-DTA and experimental selectivity estimates were highly correlated. In contrast, correlations between DrugForm-TAS predictions and experimental selectivity were consistently lower.

Comparison of the threshold-agnostic DrugForm-TAS output with its threshold-specific counterparts revealed strong correlations (Fig. 4E), indicating that the threshold-agnostic selectivity estimate is consistent with those derived using explicit affinity thresholds (7.0, 7.5, 8.0, and 8.5). Together, these correlation results demonstrate that DrugForm-TAS provides a robust estimate of ligand selectivity or, more strictly, binding propensity—comparable in quality to both DTA-based proteome-wide prediction and experimental selectivity assessments.

## Discussion

In pharmacology, “selectivity” typically describes a ligand’s ability to bind its intended target while avoiding off-target interactions at therapeutically relevant concentrations. Although DTA models address target-specific binding prediction, the DrugForm-TAS model tackles a complementary aspect: the propensity of a ligand to bind promiscuously across the proteome.

While off-target binding can, in principle, be estimated by applying a DTA model to hundreds or thousands of proteins, this approach entails prohibitive computational costs. DrugForm-TAS circumvents this bottleneck by providing a rapid, single-pass estimate of proteome-wide binding propensity—requiring only the specification of an affinity threshold, not a target protein. However, during de novo ligand generation, predicted affinities often vary widely across targets, making a threshold-agnostic selectivity metric more practical. DrugForm-TAS fulfills this need by delivering a unified selectivity score that does not depend on any predefined threshold.

Here, we introduce the concept of TAS—a holistic assessment of ligand selectivity that does not depend on a predefined target protein or a single affinity threshold but instead integrates selectivity scores across 4 thresholds (7.0, 7.5, 8.0, and 8.5). This approach enables selectivity to be treated as a global property reflecting a molecule’s overall capacity for selective binding. Applying the DrugForm-TAS model to large-scale virtual screening of chemical libraries will substantially accelerate in silico experiments as a global property reflecting a molecule’s overall capacity for selective binding. Furthermore, DrugForm-TAS can be integrated into de novo molecular design pipelines: generative models leveraging reinforcement learning can incorporate real-time feedback from DrugForm-TAS to bias sampling toward highly selective ligands.

Users should keep in mind that TAS predicts binding breadth but does not identify which proteins are bound, so the full off-target identification still requires protein-by-protein analysis. A typical use case scenario of the model may include both DTA estimation of efficiency against protein of interest, with simultaneous TAS estimating proteome-wide binding propensity, which behaves as a nonspecificity binding filter. Calculating both DTA and TAS values allows us to select those ligands that have high affinity to the target and low off-target binding probability. After the generation or library screening is completed, the full proteome DTA calculation may be performed afterward for few top-score ligands.

We performed a case study on 3 well-established therapeutic targets with extensive clinical validation: tyrosine-protein kinase JAK1 and GTPase KRas—targets of anti-inflammatory and anticancer agents, respectively—and prothrombin (coagulation factor II), a target of anticoagulant drugs. The goal was to compare the off-target binding profiles of de novo generated ligands under 3 design scenarios: (a) using threshold-specific selectivity predictions from DrugForm-TAS, (b) using only the threshold-agnostic TAS score, and (c) without any selectivity guidance. The ligand generation protocol and a quantitative comparison of predicted off-target interactions across the 3 strategies are provided in the Supplementary Materials for all 3 targets. Figures S1 to S3 demonstrate that incorporating DrugForm-TAS into the generative process yielded ligands with high target affinity and substantially fewer predicted off-target interactions compared to the unguided approach.

Thus, integrating the selectivity model into de novo ligand design eliminates the need for additional filtering to identify selective candidates after generation and initial scoring. Instead, the generative process inherently prioritizes ligands with higher selectivity for the intended target—regardless of the specific target chosen. The model is also applicable to large-scale virtual screening of existing compound libraries. However, its effective deployment requires concurrent evaluation of target affinity, which is achieved by coupling DrugForm-TAS with a DTA model.

To evaluate the performance of DrugForm-TAS, we compared 3 strategies for assessing ligand binding propensity. The most accurate but experimentally intensive approach involves in vitro or in vivo affinity measurements. However, experimental profiling is typically limited to a small subset of proteins and therefore cannot provide a comprehensive map of off-target interactions.

A second approach estimates selectivity by performing proteome-wide affinity predictions using a DTA model. Although this method offers broader coverage, it suffers from reduced accuracy due to both model limitations and noise in the experimental data used for DTA training. Moreover, its computational cost is prohibitive: generating our training dataset required approximately 2 months of computation on 12 NVIDIA A100 GPUs.

In contrast, DrugForm-TAS provides a selectivity estimate in a fraction of the time (0.365 s for 100 molecules)—5.5 thousand times faster than a single DTA prediction by design (~34 min for 100 molecules). This efficiency comes at the cost of a modest reduction in accuracy relative to full DTA-based selectivity assessment. Nevertheless, empirical validation shows that DrugForm-TAS achieves sufficient predictive fidelity to guide the generation of ligands with minimal off-target activity.

In addition, we conducted a correlation test comparing 3 distinct selectivity estimation methods. DrugForm-TAS provides the fastest but least accurate estimate. DrugForm-DTA is extremely more computationally demanding yet exhibits a lower error rate, whereas experimental estimates (Exp) rely directly on measured data but yield sparse and biased coverage limited to a small subset of well-studied ligands. The DTA–TAS correlation quantifies the fidelity of the TAS model, reflecting information loss due to compression and knowledge distillation. The DTA–Exp correlation measures the agreement between experimental measurements and DTA predictions, accounting for the substantial sparsity and bias inherent in the experimental estimates. Although Exp is derived from empirical data, it remains imperfect due to uneven coverage of the proteome and ligand space. The sparsity and bias of experimental values in BindingDB are discussed in the corresponding Supplementary Materials section.

The TAS–Exp correlation is of primary interest, as it reflects the extent to which TAS predictions are empirically supported. Lower TAS–Exp correlation values were observed compared to those of both DTA–Exp and DTA–TAS—a result that is entirely expected, given that TAS represents a second-order approximation of experimental data and thus inherits both the inaccuracies of the DTA model and the imperfections introduced during distillation. Nevertheless, as demonstrated in our generative experiments, the predictive quality of the model is sufficient to effectively filter out ligands with low target-binding propensity.

It is worth mentioning that the training split of the DrugForm-TAS model does not follow the training split of the parent DTA model. Despite concerns about optimistic test results, we consider them noncritical for the following reasons: It may affect model training metrics such as ROC-AUC or balanced accuracy, but we do not regard them as primary model success metrics. The correlation test dataset was also formed without taking into account how the DTA training dataset was split; otherwise, it would be impossible to create such a statistically reasonable dataset from a small DTA test subset. This does not affect the correlation test conclusions, because the Exp–TAS metric is calculated directly from experimental data, bypassing the DTA–TAS chain. Summing up, even if it would be more accurate to train and test the TAS model according to the DTA model split, it is constrained by the harsh reality of the deficit of uniform experimental data.

Note that DrugForm-TAS is not a direct replacement for experimental selectivity measurements or detailed off-target profiling. It is an efficient, low-cost internal consistency filter tightly coupled to the companion DTA model for prioritizing compounds for deeper analysis. Also, a high TAS score may reflect either a selective ligand or a molecule with low affinity across the proteins. Conversely, a low TAS score clearly indicates promiscuous binding to multiple proteins. We discuss this issue more closely in the corresponding Supplementary Materials section, where we compare model behavior for a highly selective ligand and for a ligand with low selectivity and a nonspecific affinity profile.

Thus, DrugForm-TAS is best interpreted as a predictor of nonselectivity rather than selectivity itself. For effective application, DrugForm-TAS must be used in conjunction with a DTA model: only when high TAS selectivity corresponds with strong predicted affinity for one or more intended targets can a ligand be considered high-quality. It should also be noted that, like the DTA model, DrugForm-TAS was trained exclusively on human and mammalian proteins; consequently, its performance on proteins from other organisms, such as bacteria, is uncertain. Additional limitations follow from the Chemformer encoder: input SMILES strings must not exceed 512 tokens, and the model is restricted to small molecules.

Thus, DrugForm-TAS is well suited for rapid assessment of a molecule’s propensity to bind multiple human proteins and for preliminary evaluation of its selectivity profile. A correlation analysis comparing the 3 selectivity estimation approaches—experimental (Exp), DrugForm-DTA, and DrugForm-TAS—revealed that threshold-specific TAS predictions show stronger agreement with experimental estimates than the threshold-agnostic score. However, this improved accuracy requires additional complexity. In contrast, the threshold-agnostic selectivity estimate provides a single, universal metric that shows global binding propensity without requiring predefined affinity thresholds.

## Conclusion

The DrugForm-TAS model estimates a ligand’s selectivity—its propensity to bind promiscuously across the proteins—with minimal computational cost. The TAS model eliminates the need to specify a reference target protein for the ligand under evaluation. The model is applicable to both de novo low-molecular-weight compound design and large-scale virtual screening of chemical libraries. Evaluation on a test set demonstrated high predictive performance, and correlation analysis revealed strong agreement between DrugForm-TAS predictions and selectivity estimates derived from experimental data as well as from proteome-wide affinity predictions using the DrugForm-DTA model. The DrugForm-TAS model, its training dataset, and source code are publicly available.

## Data Availability

The data and the code accompanying this study are freely available. The code repository (https://github.com/drugform/uniqsar) contains the framework to train and benchmark models and also to launch them for inference. The repository cannot serve big files, so they are placed at https://doi.org/10.5281/zenodo.17609738. Check the readme file to get detailed usage info and instructions to reproduce the steps of this work. Follow instructions at the code repository to merge the data into your cloned repository. Among other files are the following:• The files data/selectivity/selectivity.csv and data/selectivity/selectivity_test.csv are the constructed dataset we used to train and evaluate DrugForm-TAS. • The DrugForm-TAS model itself is stored at the models/selectivity directory. • The correlation test data/selectivity/DrugForm-TAS_correlation_test_fulldata.csv file is the correlation test results for 4,298 ligands with affinity measurements for 1,769 unique human proteins, containing selectivity estimates for each ligand in the correlation dataset using 3 distinct approaches—DrugForm-TAS, DrugForm-DTA, and experimental. • The data/selectivity/supplementary_table_generations.csv file contains the molecules, generated in the practical case study, with the calculated affinities for all proteins Considering that the code and runtime environment for neural network solutions have a limited lifespan due to the deprecation and updating of libraries and drivers, we have also prepared a Docker image that contains all components necessary for practical use of the DrugForm-TAS and DrugForm-DTA models. The image contains the trained models themselves, all software libraries, model code, and the data necessary for training and testing. Information on using the Docker image is available at https://hub.docker.com/repository/docker/drugform/uniqsar_standalone/. To download the image, use the following command: docker pull drugform/uniqsar_standalone:1.0.
