# Supplementary material for: DrugForm-TAS: Target-Agnostic Selectivity as Proteome-wide Binding Propensity Estimation
Source: Comput Struct Biotechnol J. 2026 May 18;35(1):0034. doi: 10.34133/csbj.0034 (PMC13181170; doi:10.34133/csbj.0034)
Supplement: Supplementary 1 — Supplementary Text Figs. S1 to S9 [file csbj.0034.f1.zip › DrugForm-TAS (Target Agnostic Selectivity) Supplementary.pdf]

DrugForm-TAS: target agnostic selectivity model as proteome-wide binding propensity estimation
Supplementary

Generative experiments

The primary application of the DrugForm-TAS model is *de novo* generation of small molecules. We conducted practical tests of the model by generating ligands for several common targets. The generation was performed on the DrugForm platform, the full code of which has not yet been published. However, we publish all the generation results here, including the generated ligands for each target, so that the reader can evaluate the results independently. The generator is based on a reinforcement learning (RL) algorithm that implements user-defined target optimization.

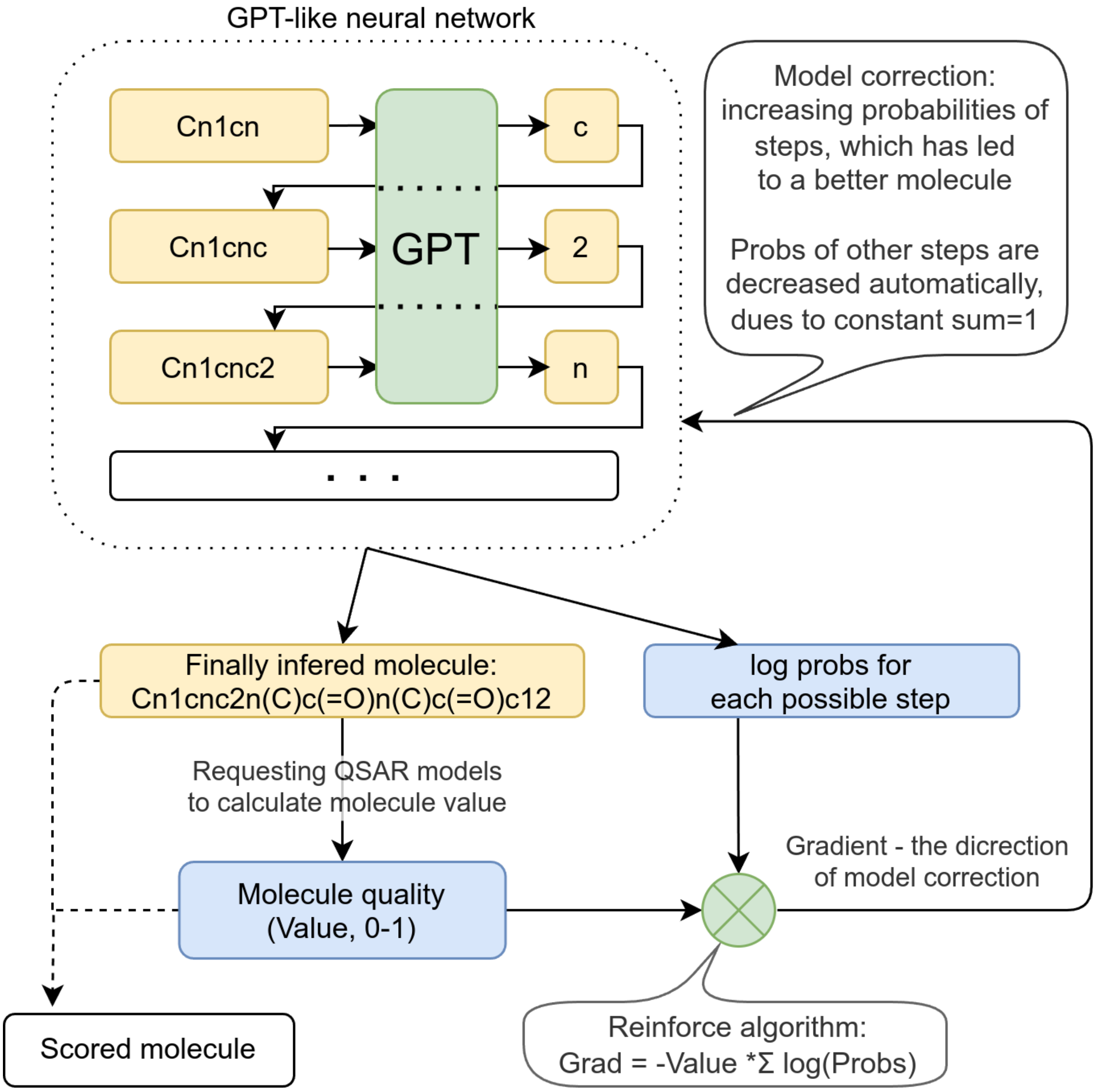

Figure S1. The schematic diagram of the generation algorithm

The molecule generation problem is formulated as a Markov decision process (MDP), in which the generative model acts as the agent, the selection of the next token in the SMILES string constitutes the agent's action, and the QSAR model serves as the environment that evaluates the trajectory by computing a reward signal. In this reinforcement learning (RL) framework, only complete trajectories (or batches thereof) are evaluated. Optimization is performed using a family of policy-based algorithms derived from the Monte Carlo Policy Gradient (MCPG) method, commonly known as Reinforce. In these algorithms, the gradient update is applied after executing a set of actions — i.e., after generating a full trajectory or batch. The gradient of the neural network weights with respect to the expected reward for a trajectory is proportional to the trajectory reward multiplied by the sum of the log-probabilities of the individual token-generation steps, thereby increasing the likelihood of actions that led to high-scoring molecules. Since the policy is normalized (i.e., the probabilities of all possible actions at each step sum to one), enhancing the probability of certain actions necessarily reduces the probabilities of alternative behaviors in a proportional manner.

To avoid premature convergence to suboptimal solutions, the algorithm incorporates stochasticity by sampling actions according to their probabilities: less probable actions can still be selected, though more probable actions are favored. On one hand, this introduces suboptimal or even chemically invalid steps, promoting exploration; on the other, it enables discovery of novel regions of chemical space. This exploration–exploitation trade-off is conveniently modulated by the temperature parameter of the Softmax function during stochastic token sampling under a Boltzmann distribution: higher temperatures increase the likelihood of selecting lower-probability (suboptimal) actions.

The generative model implements multiple variants of the Reinforce algorithm, including those with static and dynamic baselines, constraints to limit deviation from the prior molecular distribution, and multi-objective generator ensembles. During training, molecular properties and an overall composite score are computed for every generated molecule. These results are not discarded but stored in a database and ranked. Consequently, the output of the system comprises the top-ranked molecules, and the algorithm can be executed for any user-specified duration and terminated on demand once satisfactory results are obtained.

In this experiment, two generations were performed: one maximizing affinity for the target protein (DTA) and another one simultaneously maximizing affinity and minimizing the output of the threshold-agnostic selectivity model (DTA + TAS). Thus, six generations were completed, two for each target: GTPase KRas, tyrosine-protein kinase JAK1, and prothrombin (coagulation factor FII). The top 100 ligands from each generation were selected, and then the affinities for each of them to all 2,103 proteins from the list were predicted using the DTA model. We then summed the number of affinities exceeding each of the thresholds considered and presented them as separate histograms for each threshold. Supplementary figures S2–S4 show the results for each target. The grey series represents ligands obtained in the generation without the TAS model, and the colored series represents ligands obtained with the TAS model (a separate color for each threshold). As can be seen from the histograms, ligands obtained using the TAS model exhibit a substantially lower incidence of predicted off-target interactions. A list of all molecules considered, along with

the calculation results for each protein, is available in the file data/selectivity/supplementary_table_generations.csv in the repository https://github.com/drugform/uniqsar.

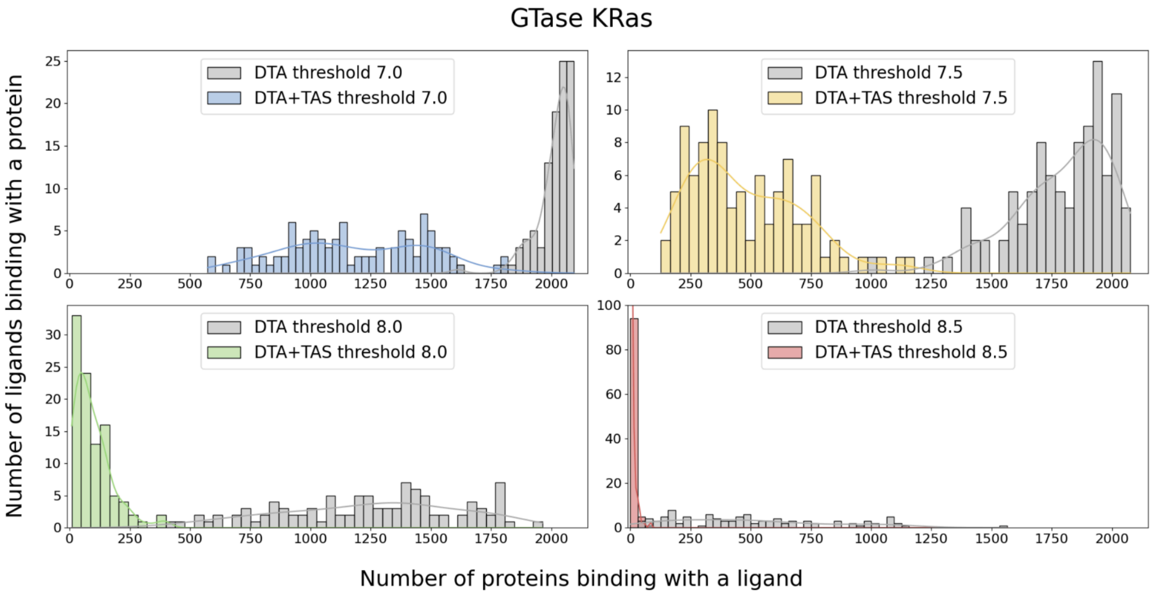

Figure S2. Comparing predicted selectivity scores of molecules, generated for the GTase KRas target, with and without the DrugForm-TAS model

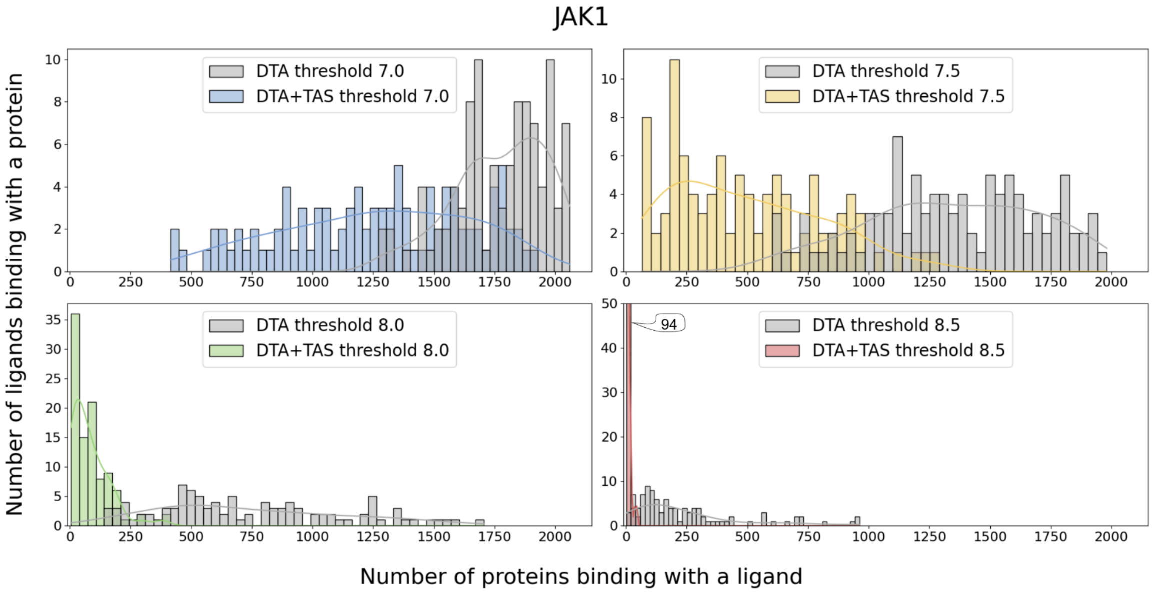

Figure S3. Comparing predicted selectivity scores of molecules, generated for the tyrosine-protein kinase JAK1 target, with and without the DrugForm-TAS model

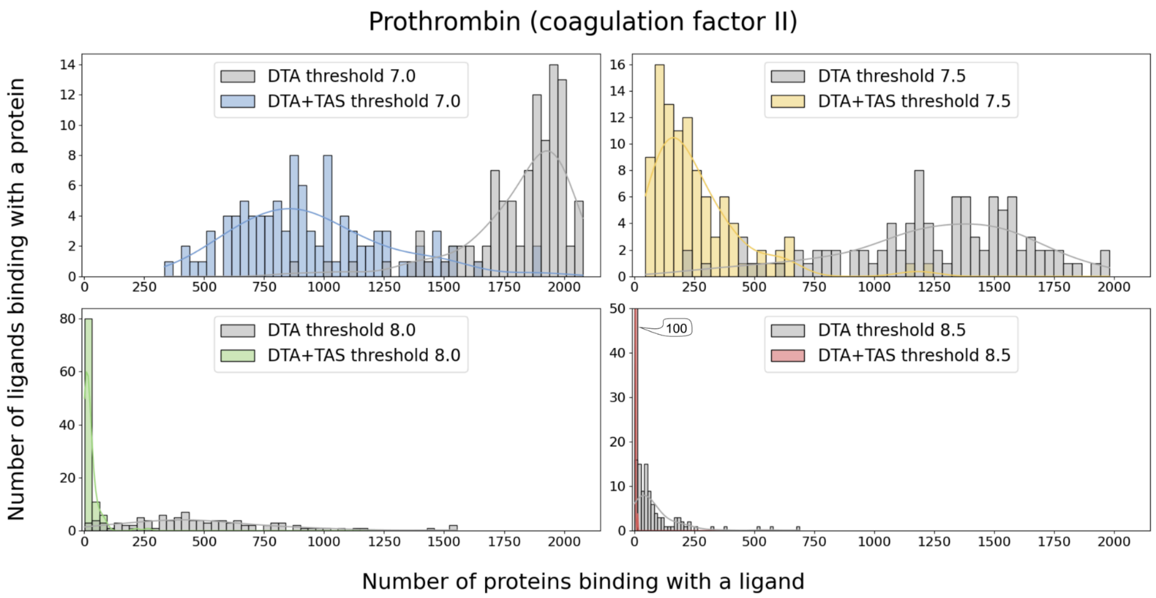

Figure S4. Comparing predicted selectivity scores of molecules, generated for the prothrombin FII target, with and without the DrugForm-TAS model

Exp selectivity estimation

The Exp selectivity estimation approach, used in the correlation test, is based on experimental values, and thus can be seen as the most reliable and ground-truth one. Figure~S4 shows the sparsity of the ligand space in the subset of 4298 ligands, selected for the correlation test. Even among ligands with many measured ligands there are only about a hundred highly investigated ligands. It means that it is possible to make such estimations only for a small number of ligands.

The Exp selectivity estimation is also biased towards a smaller subset of highly investigated proteins (Figure S5). In the selected subset two thirds of the proteome is weakly investigated. This means that even if the ligand was measured across a large variety of proteins, it does not display the proteome-wide selectivity estimation. One can speculate that if BindingDB contains a record of ligand-protein affinity, the researchers expected this ligand to have affinity with this protein, meaning that the Exp statistics is biased towards higher affinities and lower selectivities.

On the other hand, DrugForm-TAS is trained on the full set of ligands, learning the whole set of experimental measurements, and provides less biased selectivity estimates.

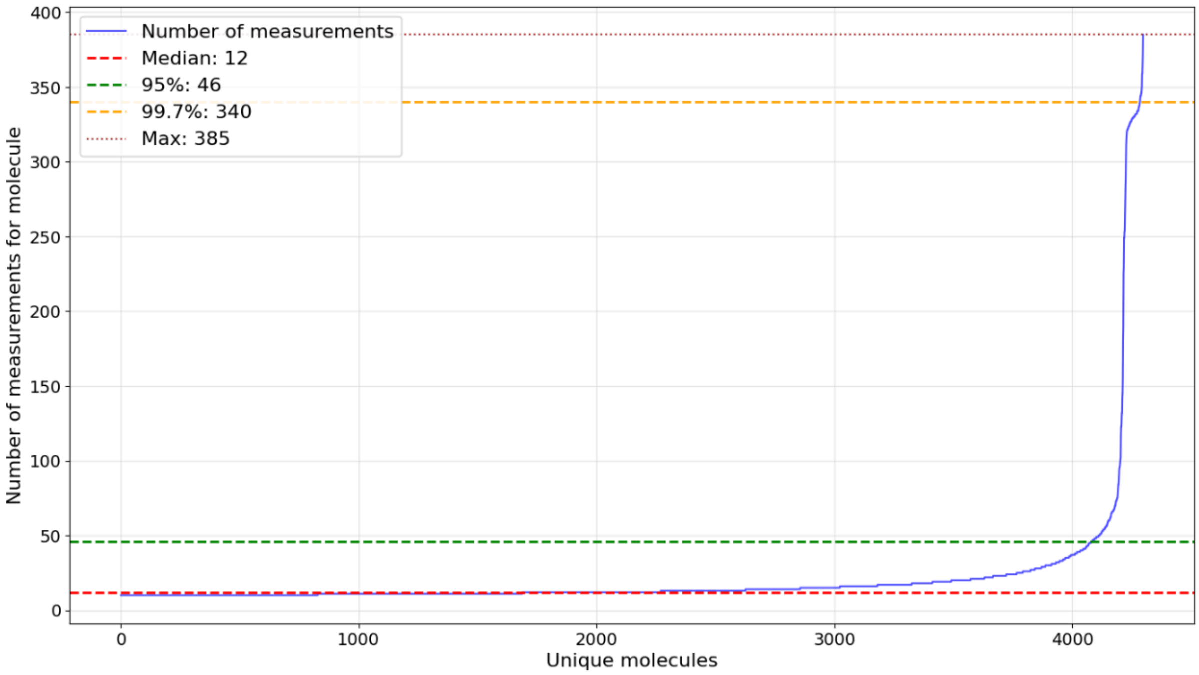

Figure S5. Sparsity of the experimentally measured ligand space.

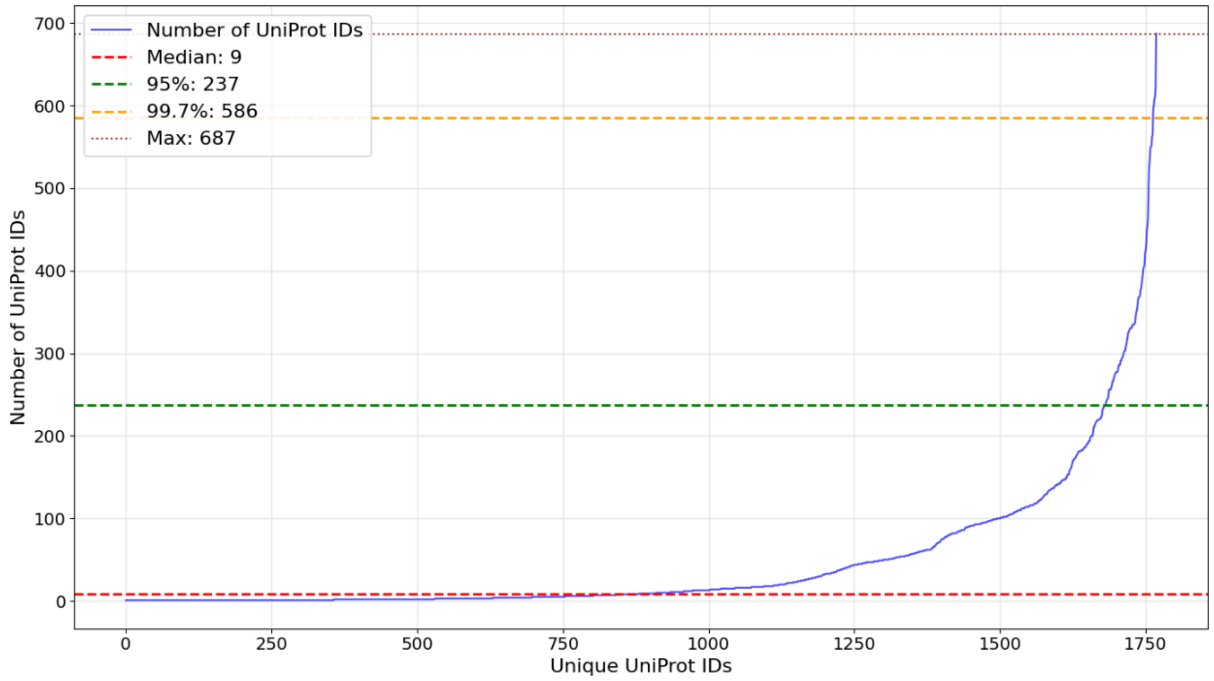

Figure S6. Bias of the experimentally measured proteome.

Calibrating binarization thresholds for model outputs

We calculated optimal binarization thresholds for model outputs:

7.0 (blue) = 0.36

7.5 (yellow) = 0.33

8.0 (green) = 0.27

8.5 (red) = 0.33

Still, the difference in F1-score compared to using simply 0.5 threshold everywhere is not critical, so one can use just 0.5 everywhere.

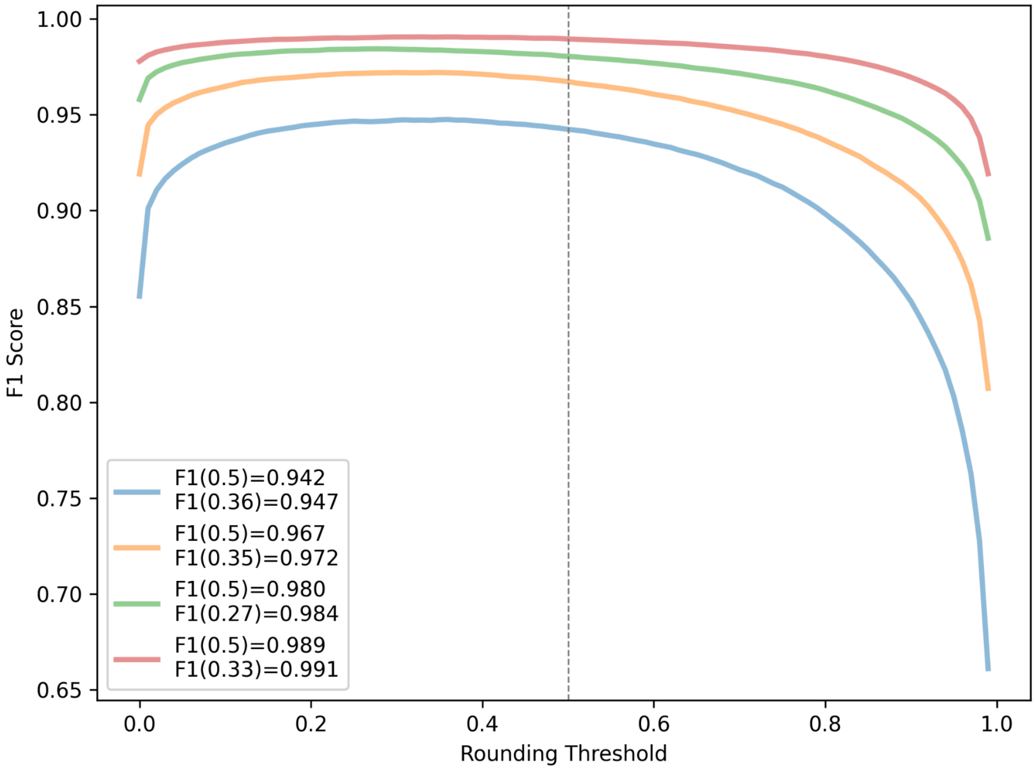

Figure S7. Calibrating binarization thresholds by maximizing F1-score for each threshold.

Selectivity versus non-specificity

The DrugForm-TAS model is positioned as a fast pre-screening tool for ligand selectivity. Strictly speaking, the model predicts ligand nonspecificity, but not selectivity, while its name reflects the actual, rather than formal, purpose of the model. As mentioned repeatedly in the article, to obtain a selectivity estimate, it must be combined with DTA model calculations for the target of interest. Without this, the model will be unable to distinguish a ligand with high selective affinity for one or more targets from a ligand with no outstanding affinity for any of them. To demonstrate this phenomenon, we selected several representative examples from the prepared BindingDB dataset with a sufficiently large number of dimensions for statistical analysis and analyzed the behavior of the TAS model on them.

Figure S8 shows the small molecule CNCCC(Oc1ccccc1C)c1ccccc1 (PubChem CID 54841), for which experimental measurements are available for 59 different targets. The sorted distribution of known values is presented in Figure S7B, which shows that this ligand can be considered highly selective towards two related targets: SLC6A2 (UniProt ID P23975) and SLC6A4 (UniProt ID P31645). The affinity model predictions (rounded to 1 decimal place) are presented in Table S7C - the value of the threshold-free TAS metric, as well as the values at all thresholds, are equal to 1.

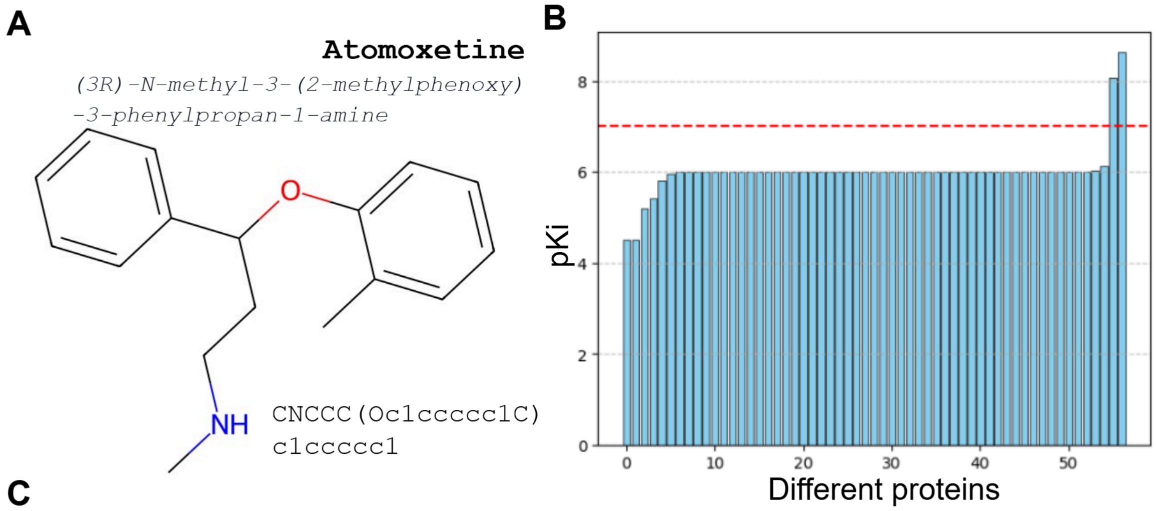

selectivity 7.0	selectivity 7.5	selectivity 8.0	selectivity 8.5	TAS
1	1	1	1	1

Figure S8. Model behaviour for a highly selective ligand.

Figure S9 shows another small molecule: `C=C(C)[C@@H]1CCC(C)=C[C@H]1c1c(O)cc(CCC)cc1O` (PubChem CID 11601669), for which 16 different targets are available for measurement. This molecule does not have significant affinity for any of the measured targets, and therefore the TAS model returns a positive prediction for it.

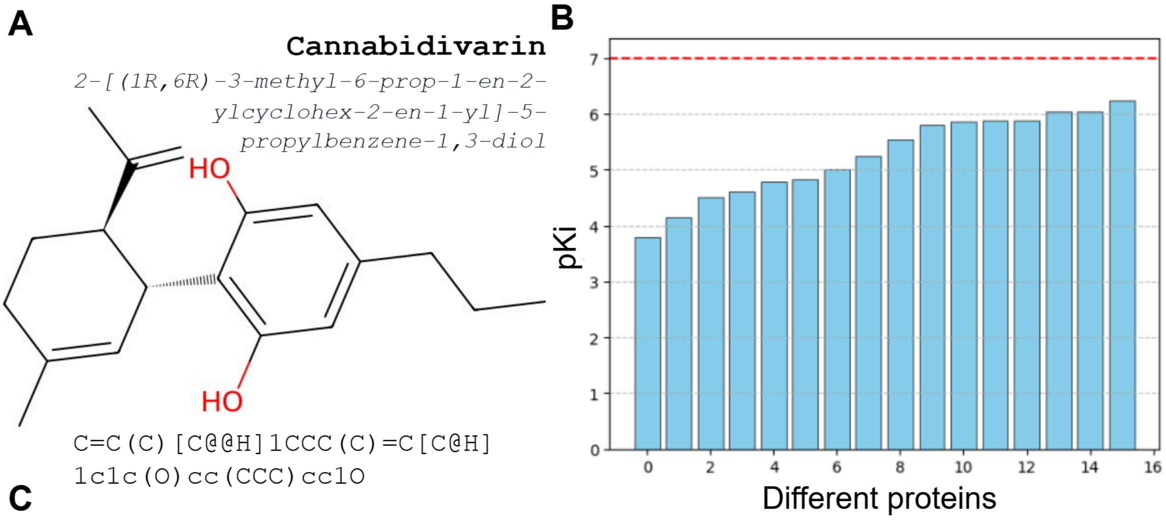

selectivity 7.0	selectivity 7.5	selectivity 8.0	selectivity 8.5	TAS
1	1	1	1	1

Figure S9. Model behaviour for a ligand with both low affinity and non-specific affinity profile.

Figure S10 shows the `COc1cc(Nc2ncc(F)c(Nc3ccc4c(n3)NC(=O)C(C)(C)O4)n2)cc(OC)c1OC` molecule (PubChem CID 11213558), which is not a complete edge case in terms of selectivity and specificity, like the two previous molecules. Looking at the sorted distribution of affinities to various proteins (Figure S10B), it can be stated that, overall, this molecule has a fairly selective, although not perfect for the threshold of 7.0, binding profile. The TAS model prediction reflects this fact: by the threshold of 7.0, the predicted value is below unity, and by all other thresholds it is equal to unity.

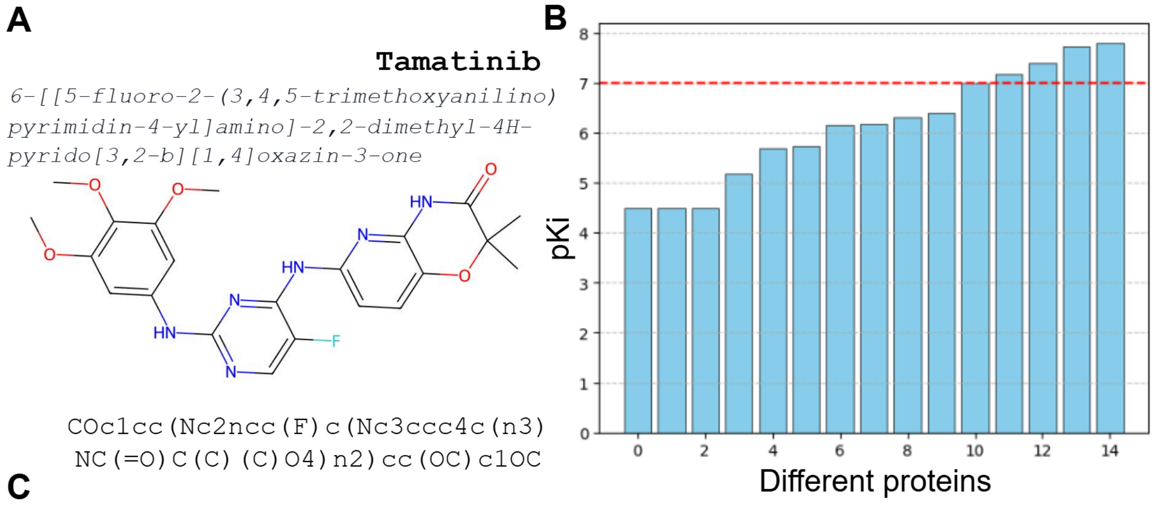

selectivity 7.0	selectivity 7.5	selectivity 8.0	selectivity 8.5	TAS
0.9	1	1	1	1

Figure S10. Model behaviour for a relatively selective ligand.
