# Supplementary figures and images for: DrugForm-TAS: Target-Agnostic Selectivity as Proteome-wide Binding Propensity Estimation
Source: Comput Struct Biotechnol J. 2026 May 18;35(1):0034. doi: 10.34133/csbj.0034 (PMC13181170; doi:10.34133/csbj.0034)

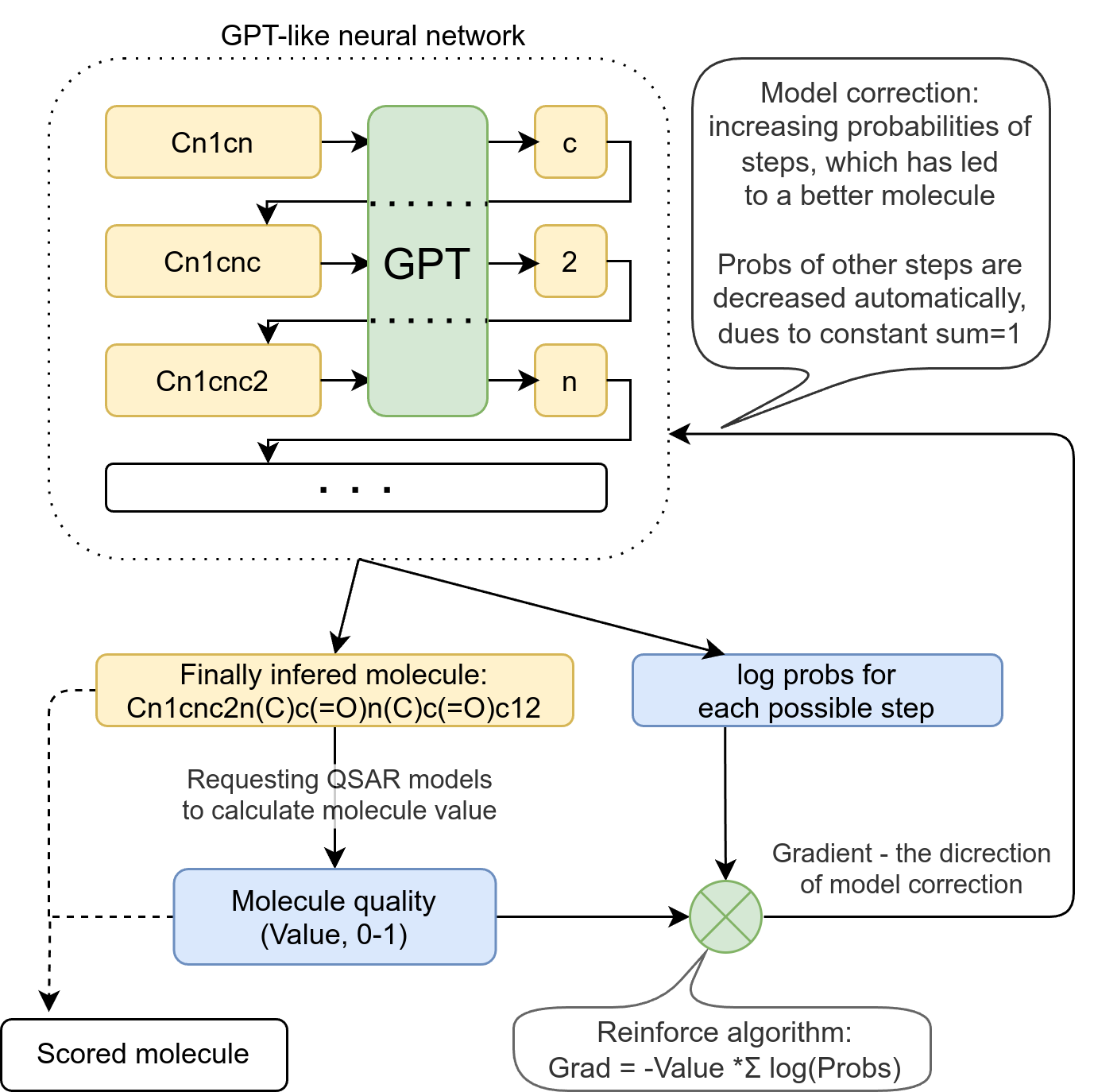

Supplement: Supplementary 1 — Supplementary Text Figs. S1 to S9 [file csbj.0034.f1.zip › Figure_S1.png]

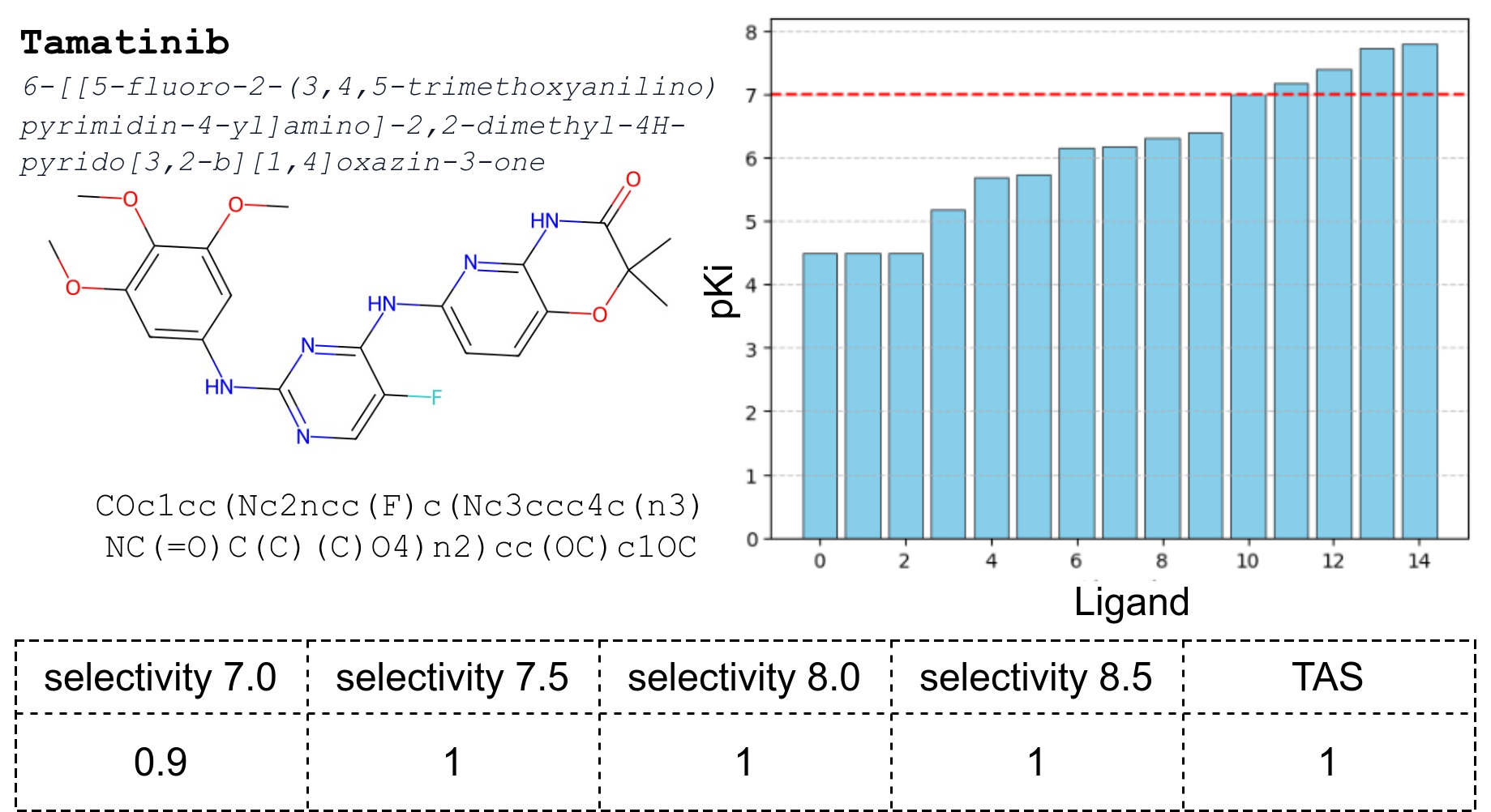

Supplement: Supplementary 1 — Supplementary Text Figs. S1 to S9 [file csbj.0034.f1.zip › Figure_S10.png]

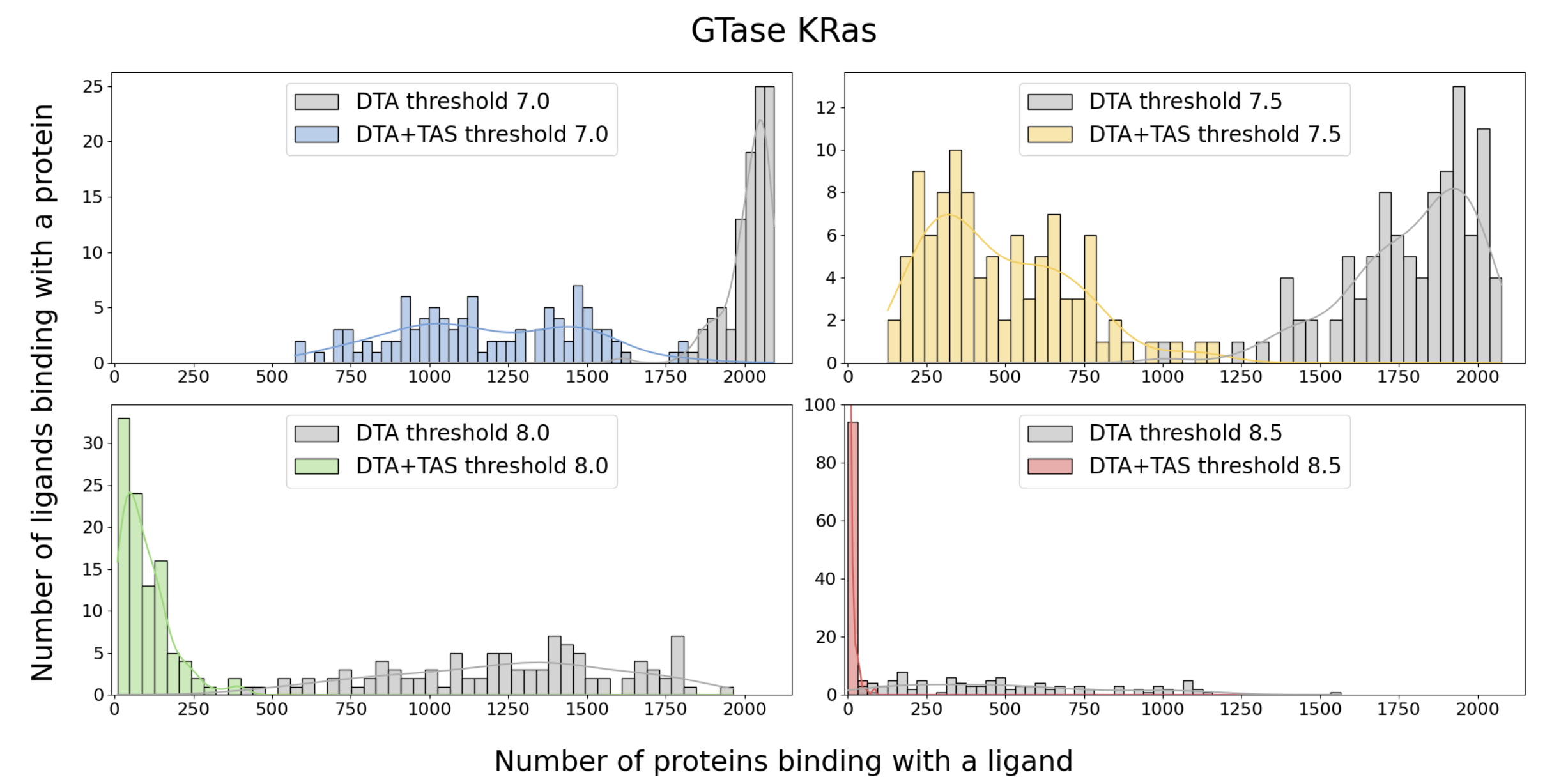

Supplement: Supplementary 1 — Supplementary Text Figs. S1 to S9 [file csbj.0034.f1.zip › Figure_S2.png]

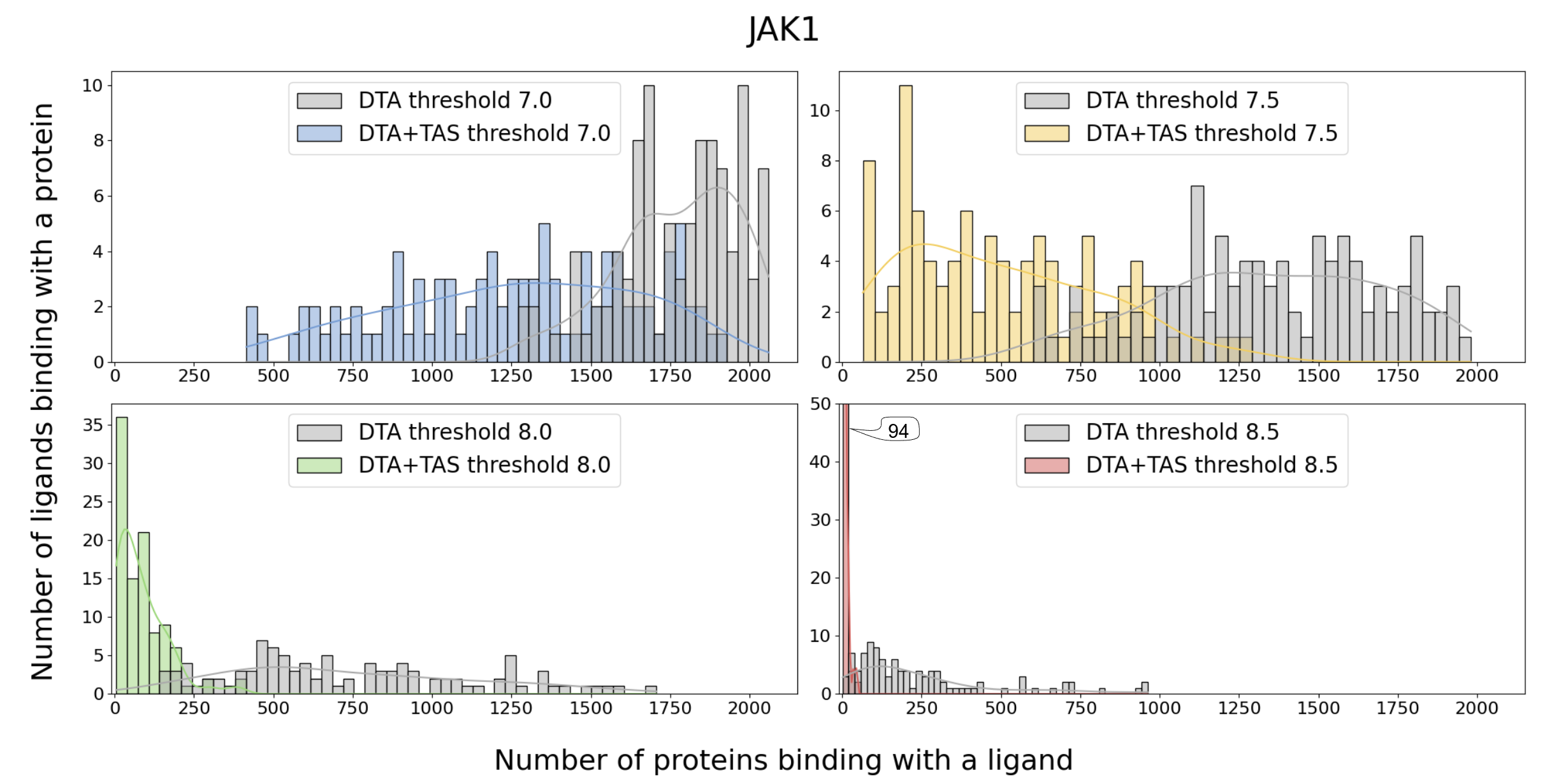

Supplement: Supplementary 1 — Supplementary Text Figs. S1 to S9 [file csbj.0034.f1.zip › Figure_S3.png]

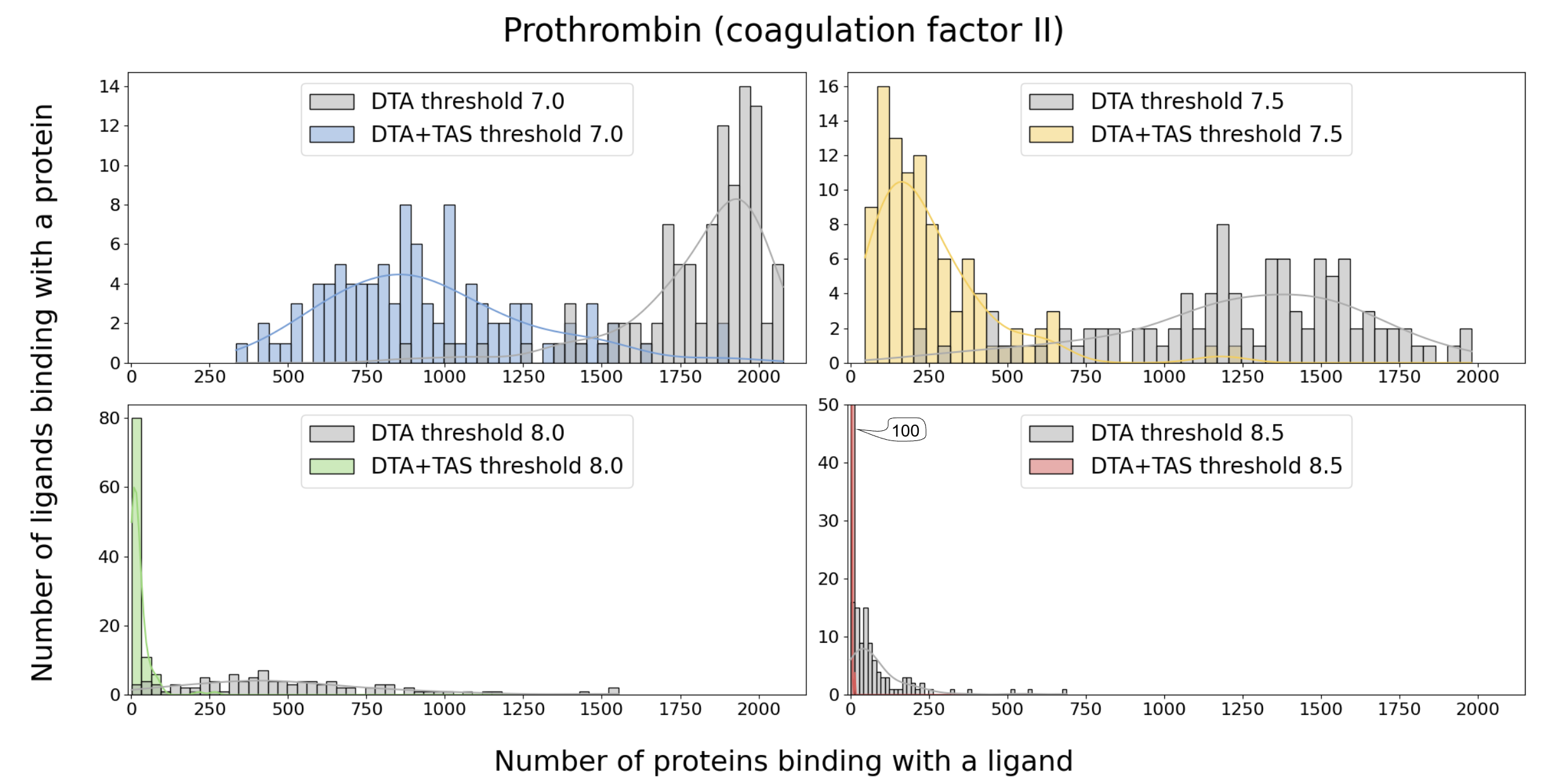

Supplement: Supplementary 1 — Supplementary Text Figs. S1 to S9 [file csbj.0034.f1.zip › Figure_S4.png]

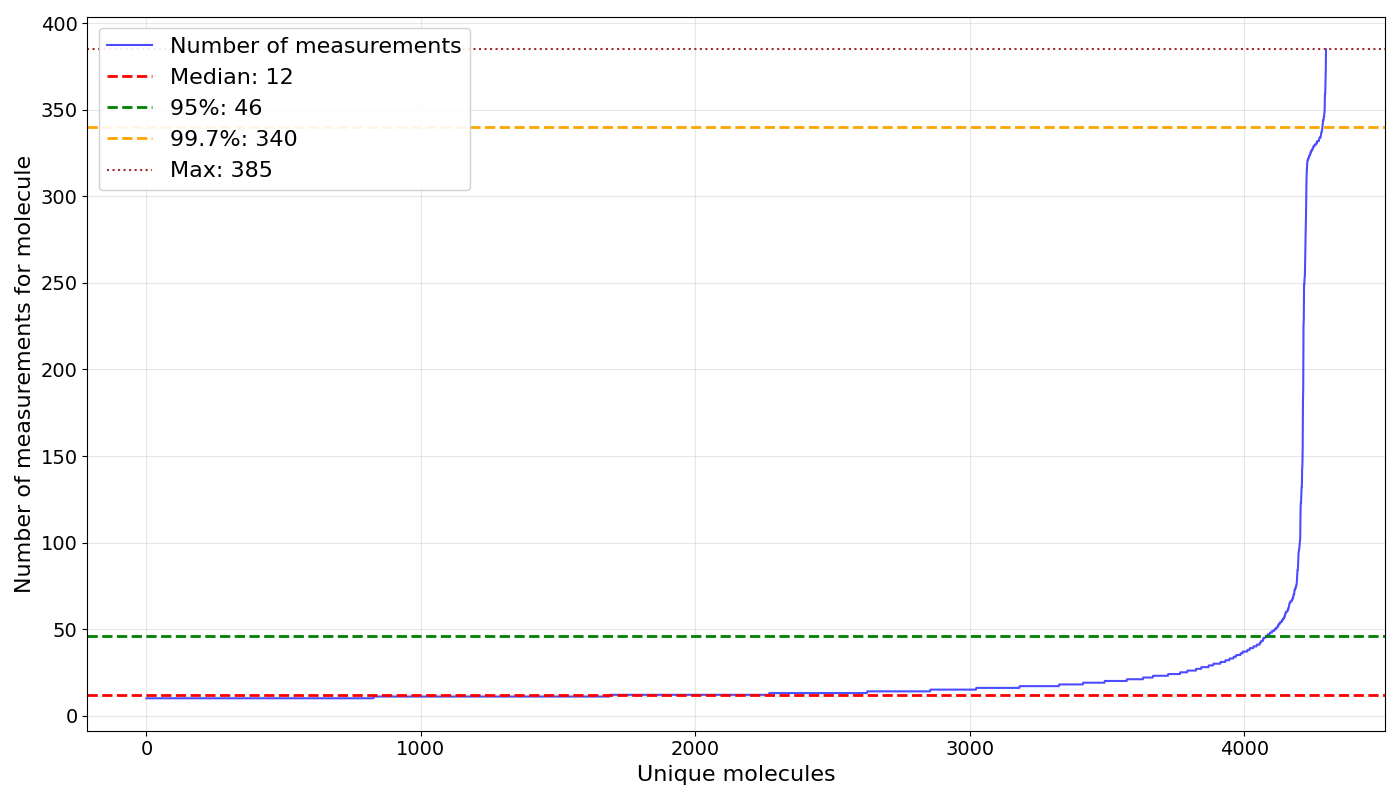

Supplement: Supplementary 1 — Supplementary Text Figs. S1 to S9 [file csbj.0034.f1.zip › Figure_S5.png]

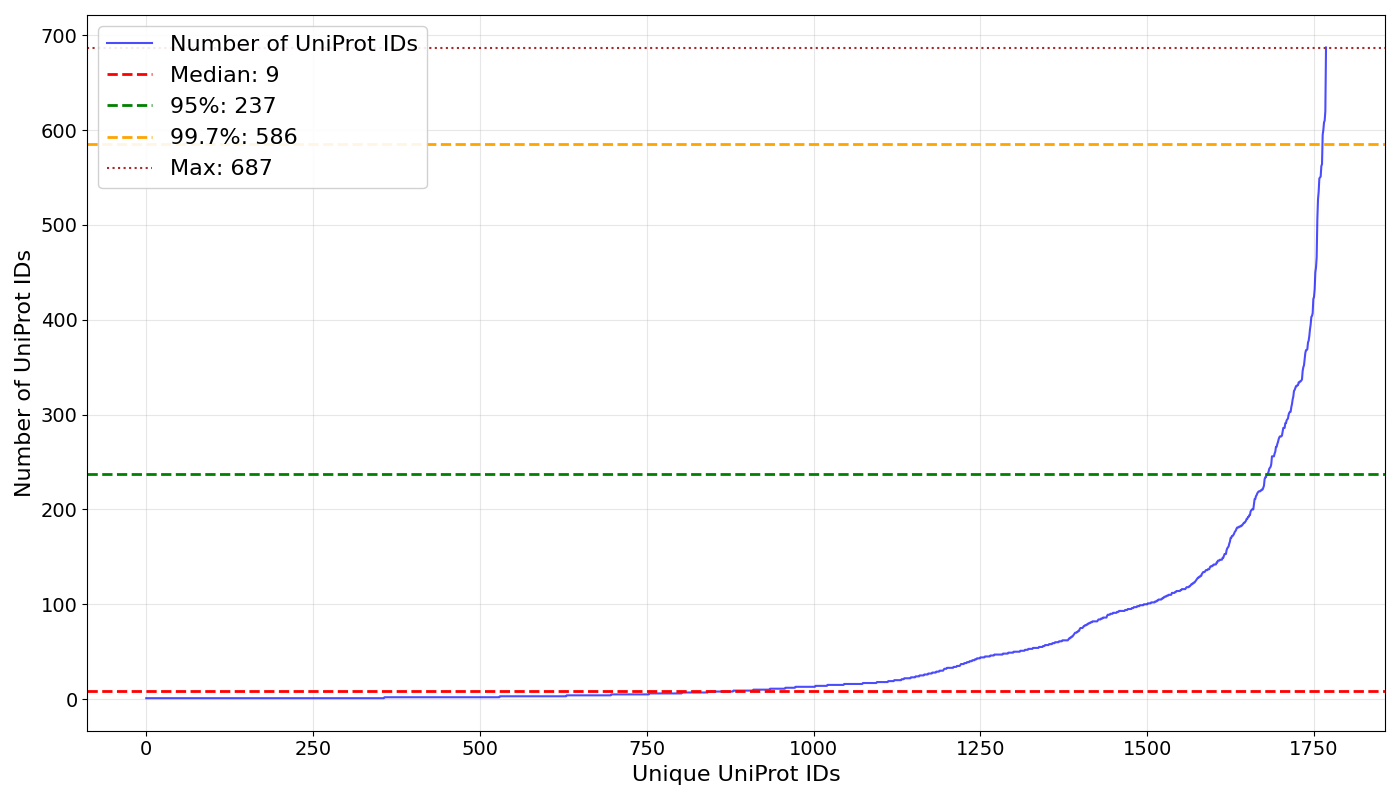

Supplement: Supplementary 1 — Supplementary Text Figs. S1 to S9 [file csbj.0034.f1.zip › Figure_S6.png]

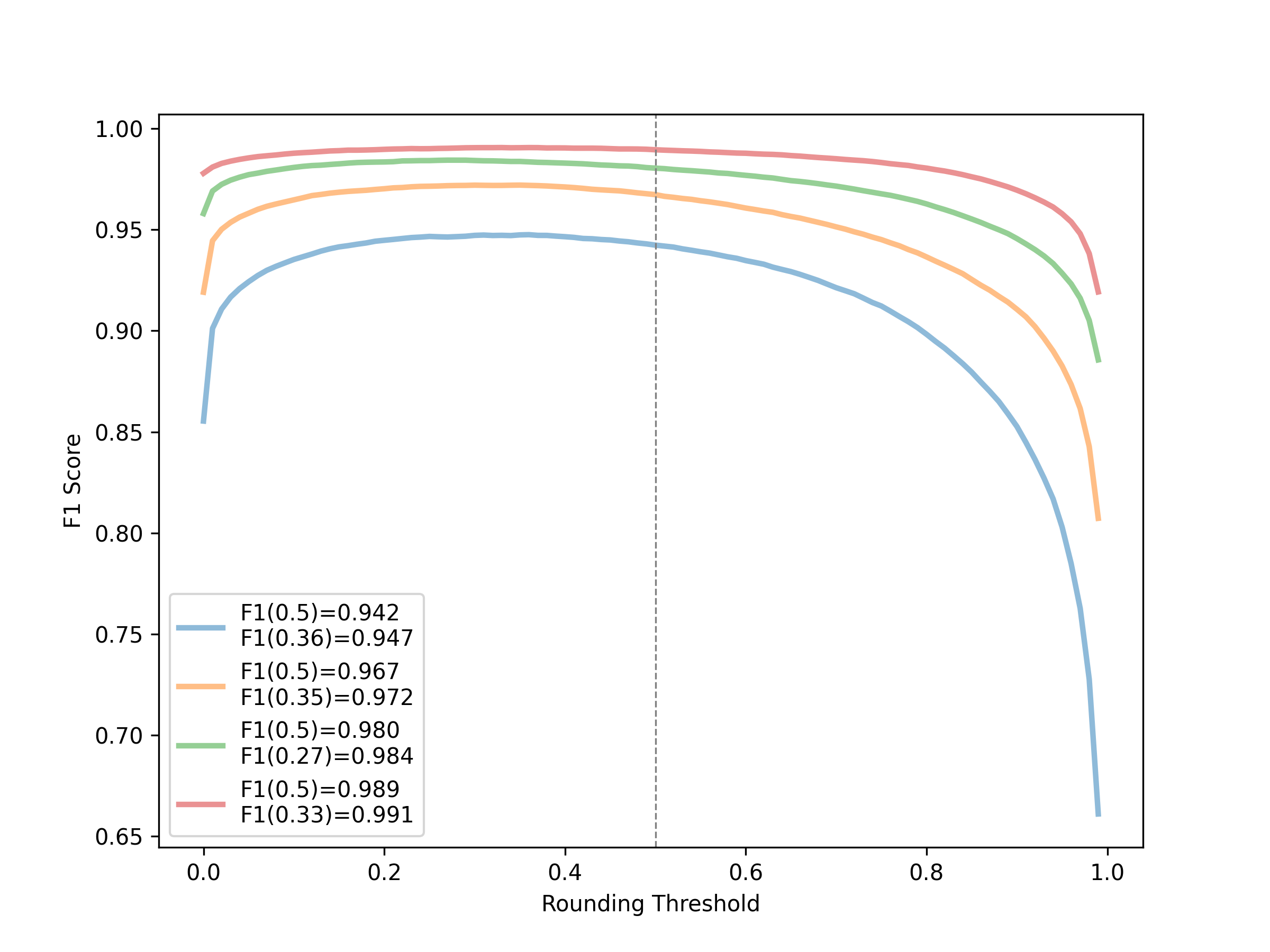

Supplement: Supplementary 1 — Supplementary Text Figs. S1 to S9 [file csbj.0034.f1.zip › Figure_S7.png]

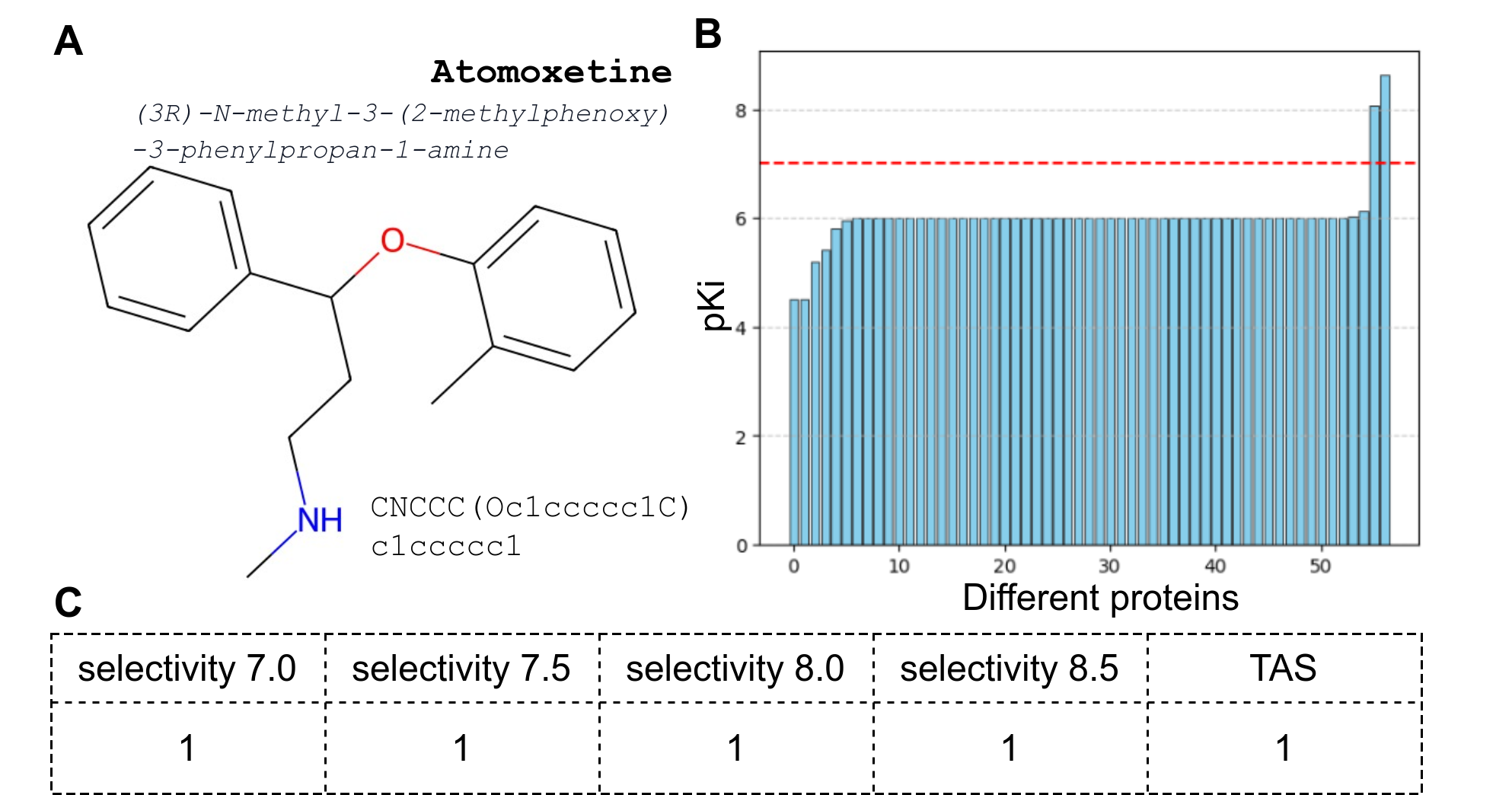

Supplement: Supplementary 1 — Supplementary Text Figs. S1 to S9 [file csbj.0034.f1.zip › Figure_S8.png]

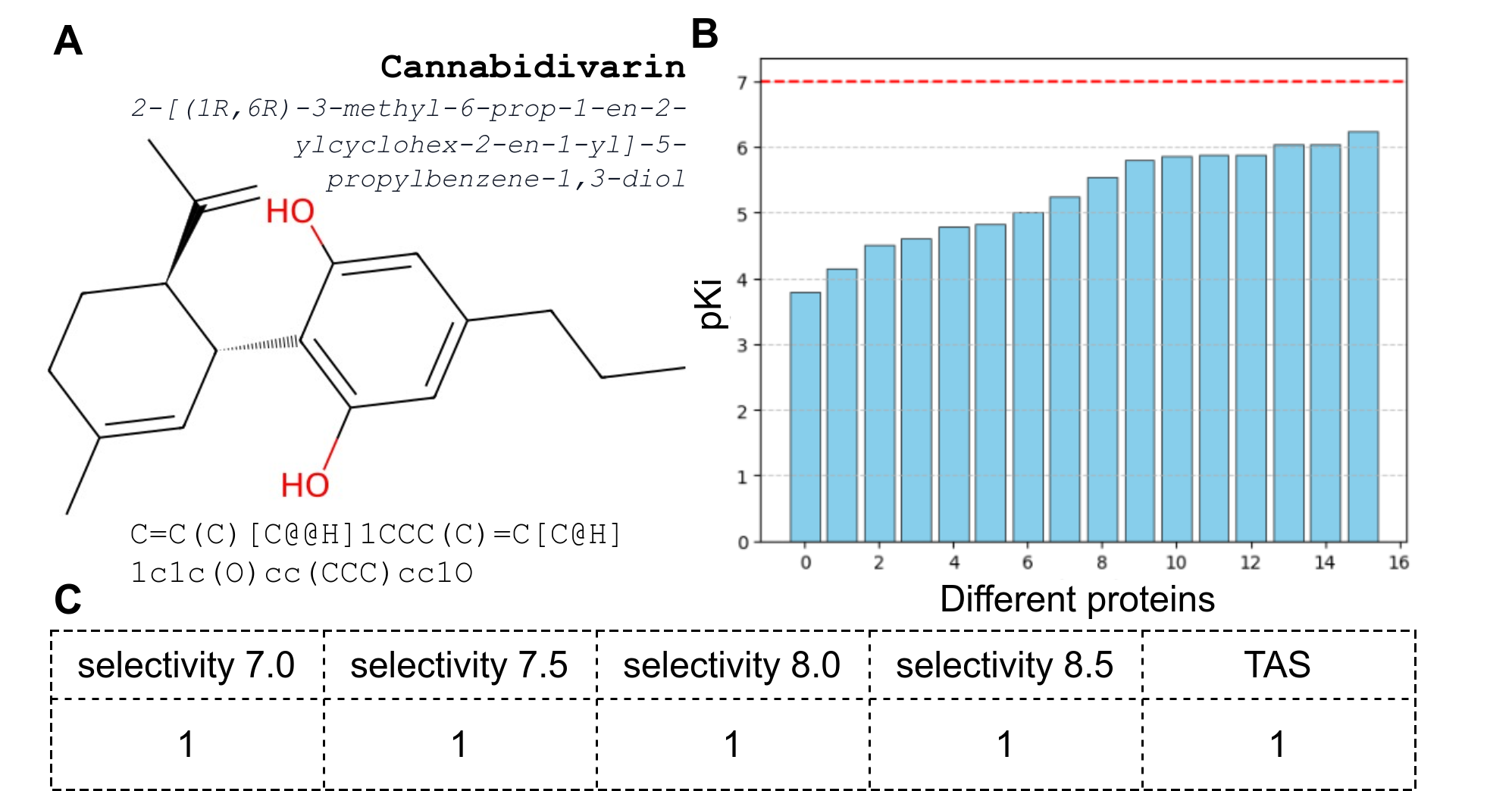

Supplement: Supplementary 1 — Supplementary Text Figs. S1 to S9 [file csbj.0034.f1.zip › Figure_S9.png]
